# Boron Nitride Reinforced Supramolecular Gels Nano‐Assembled with Fungicides toward Soil‐Borne Fungal Disease Management

**DOI:** 10.1002/advs.202515855

**Published:** 2025-11-19

**Authors:** Li Hao, Jialin Zeng, Weixian Qiu, Jinhui Wang, Xiaoshan Huang, Jiachun Wu, Zhensong Weng, Ziting Yin, Hongjun Zhou, Xinhua Zhou

**Affiliations:** ^1^ School of Chemical and Materials Engineering Key Laboratory of Green Prevention and Control on Fruits and Vegetables in South China of Ministry of Agriculture and Rural Affairs Innovative Institute for Plant Health Zhongkai University of Agriculture and Engineering Guangzhou Guangdong 510225 China

**Keywords:** antifungal efficacy, hexagonal boron nitride, nano‐assemble, soil retention, supramolecular gels

## Abstract

To overcome limitations of conventional fungicides against soil‐borne diseases like southern blight, this study develops h‐BN‐reinforced supramolecular gels for precision tebuconazole (Teb) delivery. These gels are engineered organic solvent free via one‐step amino‐amide self‐assembly with polyprotic acids. h‐BN nanosheets embed uniformly within the gel matrices with narrowing pore distributions and enhancing mechanical stability. h‐BN's hydrophobicity, affinity for the gel's carbon chains, and *π*–*π* interactions with Teb collectively restricts Teb diffusion, reducing its cumulative release rate. The gels’ inherent pH and temperature‐responsive gel‐sol transitions enable dual stimuli‐responsive sustained fungicide release. The h‐BN reinforced carriers exhibit superior interfacial properties, reducing contact angles due to h‐BN's hydrophobicity and capillary channel formation. Fluorescence after rinsing confirms significantly boosted adhesion and rain resistance versus commercial formulations. Interactions with soil are tunable by h‐BN disrupted the supramolecular structure, exposing Teb for moderate soil adsorption while extending Teb retention time (940 min, 14.69 times longer than free Teb) and enhancing root‐zone retention (41.45% vs. 13.48% for commercial). Such system also shows superior efficacy against *Sclerotium rolfsii* (*EC*_50_ reduced by 54.10%), achieving complete mycelial suppression in pot trials while promoting root growth. The nano‐assemble also reduces phytotoxicity. This work combines h‐BN's adhesive strength with supramolecular stimuli‐responsiveness for targeted, eco‐friendly disease management.

## Graphical abstract

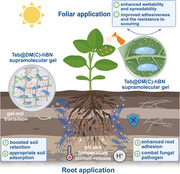

## 1 Introduction

Soil‐borne fungal pathogens such as *Sclerotium rolfsii* (southern blight) present a major challenge in precision agriculture, affecting more than 600 plant species with devastating economic consequences.^[^ ^1^, ^2^, ^3^ ^]^ The pathophysiology of this common phytopathogen involves the formation of sclerotia, metabolically quiescent, melanized survival structures that exhibit extraordinary environmental resilience owing to their dense hyphal architecture and protective exopolysaccharide matrices.^[^ ^4^, ^5^ ^]^ The treatment of these fungal pathogens needs sustained antifungal intervention at the rhizosphere‐soil interface.^[^ ^6^, ^7^, ^8^ ^]^ The underlying physicochemical challenge extends beyond simple delivery optimization, requiring control over molecular transport across heterogeneous interfaces spanning nanometer‐ to centimeter‐length scales.^[^ ^9^ ^]^ Such interventions must also achieve selective accumulation at pathogen‐plant contact zones while minimizing off‐target environmental distribution.^[^ ^10^ ^]^

Conventional fungicide delivery systems, such as those from the triazole class, encounter fundamental transport limitations, including rapid photodegradation, poor soil‐matrix penetration, and uncontrolled environmental dissipation.^[^ ^11^, ^12^, ^13^, ^14^ ^]^ The common modes of delivery, such as suspension concentrates (SC), emulsifiable concentrates (EC), rely on surfactants and organic solvents that facilitate initial dispersion but fail to anchor the fungicide at the target site, leading to rapid leaching into groundwater and diffusion away from the rhizosphere.^[^ ^15^, ^16^, ^17^ ^]^ The underlying physicochemical challenge, therefore, lies not only in protecting the active molecule but also in re‐engineering the formulation itself to achieve selective, prolonged accumulation at the pathogen‐plant‐soil interfaces, which is a problem that fundamentally requires control over molecular transport phenomena across multiple length scales.

To address the need for agriculturally adaptive systems, researchers have recently explored supramolecular gels as intelligent agrochemical delivery systems.^[^ ^18^, ^19^ ^]^ Such supramolecular materials form via the thermodynamically driven self‐assembly of low molecular weight gelators (LMWGs), which is governed by a combined effect of hydrogen bonding, electrostatic interactions, and hydrophobic desolvation.^[^ ^20^, ^21^ ^]^ This can yield the creation of fibrillar architectures capable of reversible sol‐gel transitions in response to triggers such as pH and temperature.^[^ ^22^, ^23^ ^]^ For instance, macrocycles such as cucurbit[n]urils have been used to encapsulate benzimidazole‐derived fungicides with high affinity and protection from photodegradation while the host is covalently integrated into a hydrogel backbone.^[^ ^24^, ^25^, ^26^ ^]^ In a complementary strategy, other researchers have engineered direct co‐assembly systems where anionic peptide‐based gelators electrostatically capture and structure cationic fungicides,^[^ ^27^, ^28^ ^]^ thus making the active ingredient an integral component of the gel network itself. Some other examples include Xu et al. developed a supramolecular hydrogel with a 3D porous network that adsorbed and fixed hymexazol, minimizing its migration.^[^ ^29^ ^]^ Dai et al. reported a multifunctional supramolecular complex constructed by host‐guest encapsulation of a thienyl‐engineered ingredient into *β*‐cyclodextrin with integrated architecture and reduced the bounce and splash behaviors of droplets.^[^ ^30^ ^]^

Building on these encapsulation and co‐assembly principles, recent effort has been directed toward engineering gels for stimuli‐responsive release tailored to agricultural conditions.^[^ ^31^, ^32^ ^]^ pH‐responsive gels, constructed from polymers such as chitosan or LMWGs containing carboxylic acid or amine moieties, can swell or dissolve in the slightly acidic rhizospheric microenvironment, triggering localized release of the entrapped fungicide.^[^ ^33^, ^34^ ^]^ Recently, Adams reported a study on the physical embedding of proteins by low molecular weight supramolecular gels, which can restrict the diffusion of proteins and prevent their aggregation.^[^ ^35^ ^]^ More advanced systems utilize light as a trigger, incorporating photo‐switchable units such as azobenzenes into the gelator backbone, allowing for on‐demand release with spatiotemporal precision using specific wavelengths of light, thus converting photodegradation from a liability into a control mechanism.^[^ ^36^ ^]^ Further advancements have been achieved by integrating dynamic covalent cross‐links, such as boronic esters, which create robust yet reversible networks sensitive to both pH changes and the presence of diols (e.g., sugars from root exudates).^[^ ^37^, ^38^ ^]^

Despite representing some scientific advances by these works, the state of the art in supramolecular gel‐based delivery still needs improvements in their intrinsic properties. Pristine supramolecular gels can rheologically be unstable under the shear stresses encountered in soil.^[^ ^39^ ^]^ Furthermore, their open, porous networks typically lead to uncontrolled, diffusion‐limited burst release, failing to provide the long‐term, sustained kinetics required to combat persistent pathogens such as *Sclerotium rolfsii*.^[^ ^40^, ^41^, ^42^ ^]^

The integration of 2D nanosheets into polymer composites has been widely investigated as a strategy for mechanical reinforcement.^[^ ^43^, ^44^ ^]^ Fillers such as hexagonal boron nitride (h‐BN) are known to improve the bulk mechanical properties of composites.^[^ ^45^ ^]^ Yet, this approach has predominantly treated 2D materials as passive structural fillers, largely overlooking the rich interfacial chemistry at the nano‐interface.^[^ ^46^ ^]^ The unique combination of h‐BN hydrophobic basal plane and its chemically active, polar edges presents an underexploited opportunity for multifunctional modulation. How these distinct surface domains could be used to actively participate in the thermodynamic self‐assembly of a soft matter matrix or to dynamically regulate molecular transport in response to external stimuli has remained a significant, unaddressed gap in the field of smart materials design.^[^ ^47^, ^48^ ^]^

**Scheme 1:**
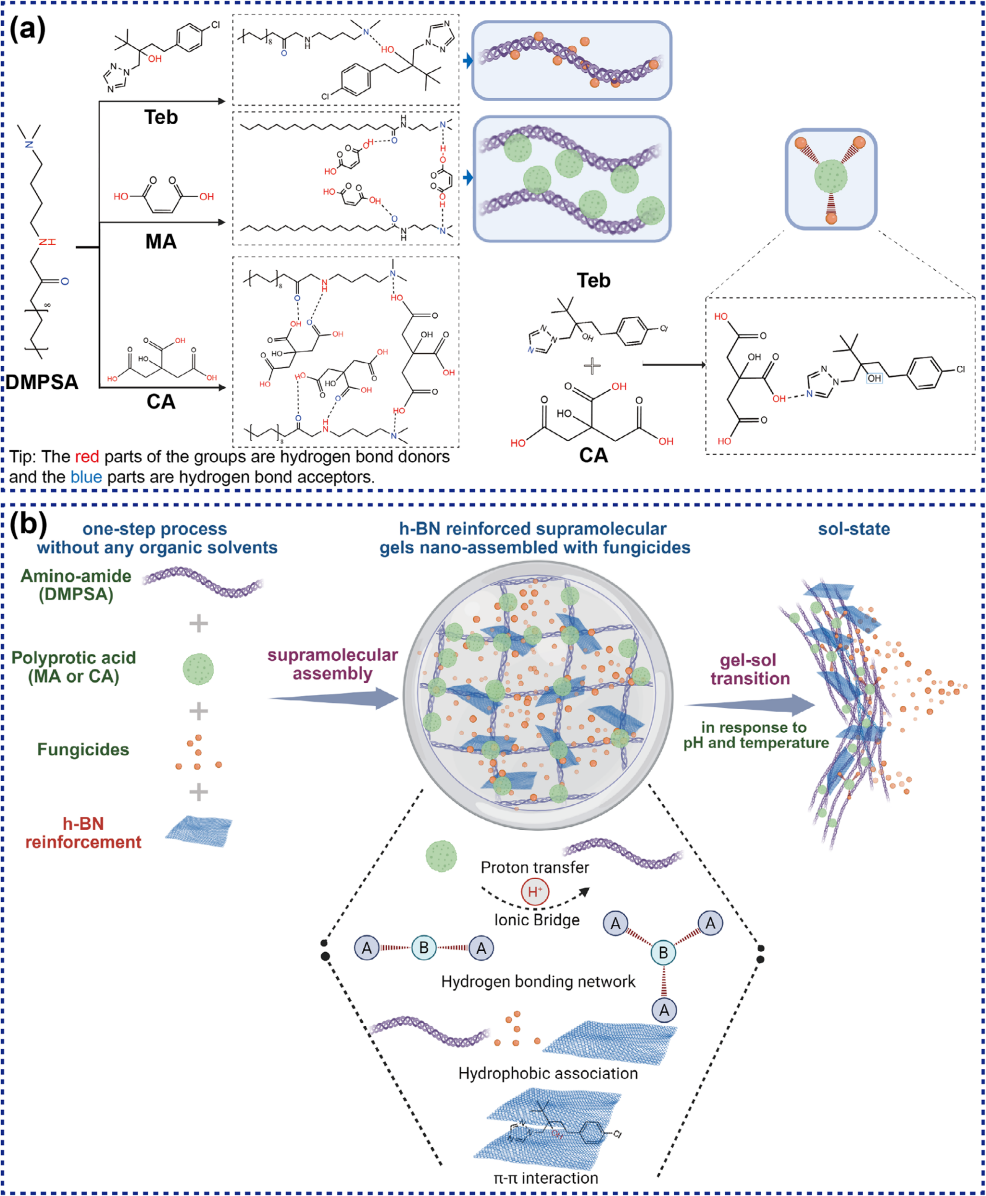
a) The supramolecular assemble process via hydrogen bond among DMPSA with MA, CA, and Teb, and the hydrogen bond between CA with Teb, b) schematic diagram of one‐step supramolecular assembly to prepare h‐BN reinforced supramolecular gels with fungicides and the behavior of gel‐sol transition in response to pH and temperature.

This paper bridges these scientific and research gaps through the design and characterization of a new class of hybrid material: a stimuli‐responsive supramolecular gel created via a one‐pot co‐assembly of a kind of amino‐amide, i.e. *N*‐[3‐(dimethylamino)propyl]stearamide (DMPSA), a polyprotic acid (MA or CA), the fungicide tebuconazole, and 2D h‐BN nanosheets, as described in **Scheme** **1**. In this system, the h‐BN nanosheet functions not as a passive inclusion but as an active, integral component of the architecture and function of the material. Through its multivalent surface, h‐BN simultaneously engages in π‐π stacking with the aromatic fungicide, hydrophobic anchoring within the alkyl chains of the gelator, and hydrogen bonding with the polyprotic acid via its edge sites. This represents the first demonstration of 2D material‐mediated stimuli‐responsive transport in soft matter systems, opening new avenues for designing adaptive materials with programmable permeability characteristics. Such multifunctional interaction establishes new topological constraints that re‐engineer the free energy landscape of the gel, leading to a hybrid material with superior mechanical integrity and emergent adhesive properties at plant‐soil interfaces.

The rationale selection of the amino‐amide DMPSA gelator represents a departure from conventional bis‐urea or peptide‐based agricultural gelators, relying on its unique ability to form both protonation‐dependent electrostatic networks and directional hydrogen‐bonded assemblies.^[^ ^49^, ^50^ ^]^ The molecular architecture of DMPSA having a dimethylamino terminus (pKa ∼ 9.2) and dual amide linkages separated by a methylene spacer enables pH‐triggered switching between neutral (self‐complementary H‐bonding) and cationic (electrostatic cross‐linking) states, a bistability absent in monofunctional gelators.^[^ ^51^, ^52^ ^]^ The incorporation of polyprotic acids (maleic acid with pKa_1_ = 1.9, pKa_2_ = 6.1; citric acid with pKa_1_ = 3.1, pKa_2_ = 4.8, pKa_3_ = 6.4) serves a dual function. This process establishes a buffered pH gradient within the gel matrix that spatially modulates DMPSA protonation states,^[^ ^53^ ^]^ and provides multiple carboxylate anchoring sites for coordination with soil cations (Ca^2^⁺, Mg^2^⁺, Fe^3^⁺).^[^ ^54^ ^]^ This polyprotic acid selection criterion specifically targets the pH range 4.5–7.0 characteristic of agricultural soils, where partial deprotonation creates amphiprotic species capable of mediating gelator‐gelator interactions through asymmetric salt bridges. Importantly, tebuconazole incorporation relies on its triazole nitrogen (pKa ∼ 5.0) and chlorophenyl moiety to participate in the supramolecular network through both hydrogen bond acceptance and *π*–*π* stacking (interplanar distance ≈3.4–3.6 Å), transforming the fungicide from a passive cargo to an active network component. Namely, this systems as a whole intrinsically couples drug (active ingredient) release to network disassembly rather than simple diffusion. This multicomponent approach generates a frustrated supramolecular landscape with competing interaction modes (ionic vs. H‐bonding vs. aromatic) that, while creating the assembly challenges discussed earlier, also provides multiple orthogonal triggers for environmental responsiveness.

**Scheme 2:**
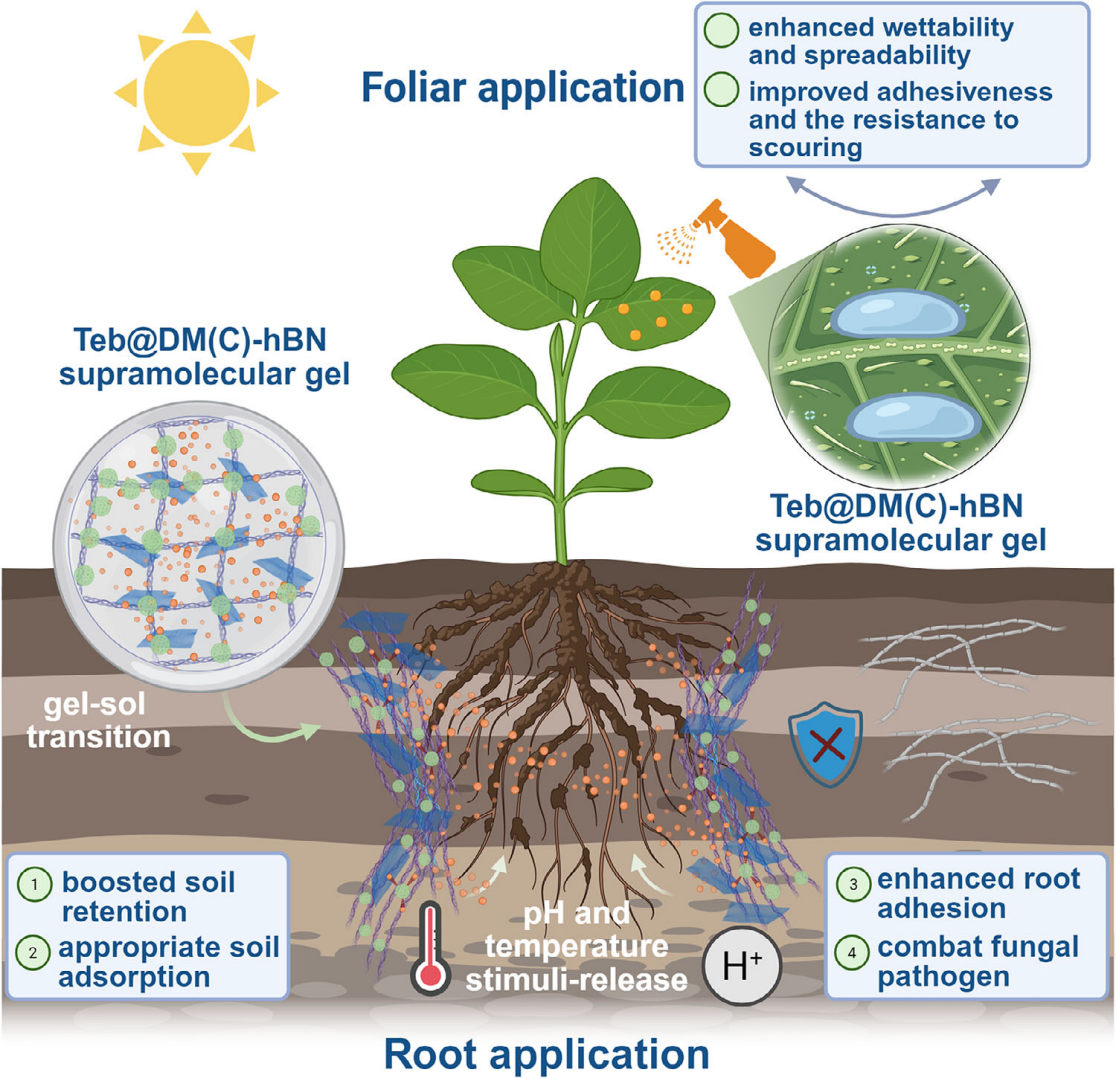
Schematic diagram of application scenarios and advantages of h‐BN reinforced supramolecular gels nano‐assembled with fungicides (Teb@DM‐hBN and Teb@DC‐hBN).

The fundamental contribution of this work is the experimental elucidation of these synergistic effects and the underlying physicochemical phenomena. We demonstrate for the first time that h‐BN nanosheets act as thermodynamic and kinetic modulators within a supramolecular agrochemical gel. Our findings reveal a novel, dual‐stimuli responsive (pH and temperature) release mechanism where the disassembly of the gel network is coupled with the physical diffusion barriers imposed by the nanosheets, a transport behavior that deviates from classical models. Furthermore, we establish a direct link between the hierarchical nano‐architecture of the material and its macroscopic performance. The system exhibits unusual properties, including enhanced adhesion, improved soil persistence, and reduced leaching‐off, which are shown to translate directly to superior, long‐term antifungal efficacy and reduced environmental toxicity in soil column and pot trial experiments, as illustrated in **Scheme** **2**. This study therefore provides not only a highly effective solution for managing soil‐borne fungal disease but also establishes a novel, adaptive materials where the functionalities of adaptive, viscoelastic soft matter and hard, 2D nanomaterials are synergistically combined.

## 2 Results and Discussion

### 2.1 Morphological and Structural Characterization

**Figure 1:**
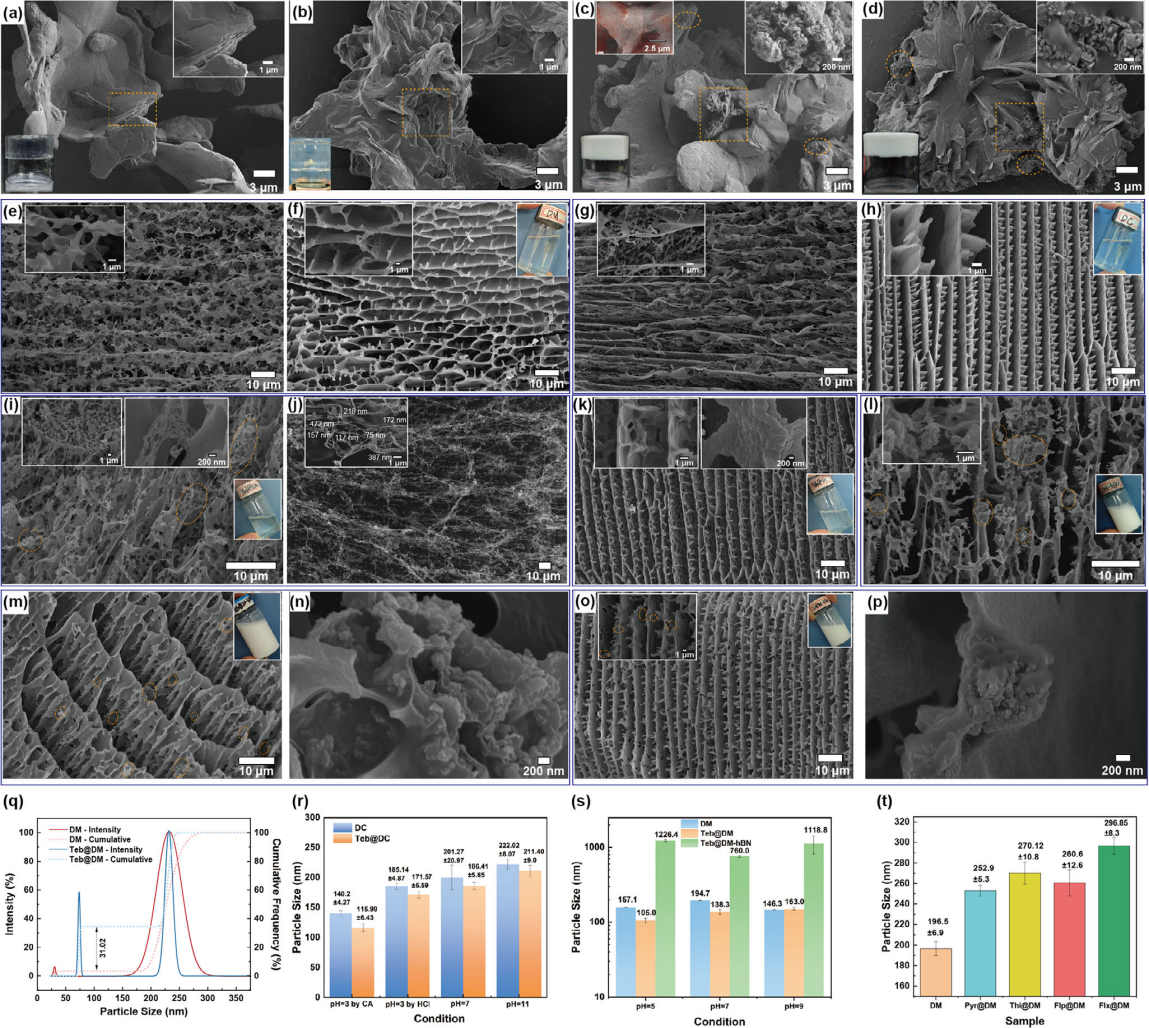
SEM images of freeze‐dried supramolecular gel: a) Teb@DM, b) Teb@DC, c) Teb@DM‐hBN with inset of Cl element mapping, d) Teb@DC‐hBN, where left corner insets displaying digital photos of corresponding inverted gel state supramolecules and right corner insets showing corresponding SEM images with higher magnifications. Cryo‐SEM images of sol‐state supramolecules: DM with DMPSA and MA ratio of e) 2:1and f) 2:1.5, DC with DMPSA and CA ratio of g) 2:1and h) 2:1.5, i) Teb@DM, j) Teb@DM under diluted 100 times condition, k) Teb@DC, l) DM‐hBN, m) Teb@DM‐hBN, n) local magnification of Teb@DM‐hBN, o) Teb@DC‐hBN, p) local magnification of Teb@DC‐hBN, where insets exhibiting higher magnifications and displaying digital photos of corresponding sol state supramolecules. q) Particle size distribution and cumulative frequency curves of DM and Teb@DM, r) particle size of DC and Teb@DC under different pH conditions, s) particle size of DM, Teb@DM, and Teb@DM‐hBN under different pH conditions, and t) particle size of DM assembled with other fungicides.

SEM revealed the microstructure of freeze‐dried supramolecular gels. Teb@DM gel exhibits a parallel‐aligned lamellar structure whose wrinkled textures originate from solvent sublimation during freeze‐drying (**Figure** **1**a). Compared to the freeze‐dried DM gel (Figure S2b, Supporting Information), the assemble of Teb within DM leads to tighter stacking between lamellae, forming a more uniform and ordered layered structure. This suggests that Teb molecules act as bridging agents during self‐assembly, providing additional stability, reducing structural defects, and enhancing interlayer interactions, thereby improving structural compactness and order. The uniform distribution of Teb within the DM matrix evidenced by the smooth and homogeneous lamellar surfaces without protruding nodules and uniform Cl element mapping indicates good compatibility between Teb and DM, rather than mere physical mixing. In contrast, h‐BN nanosheets primarily relies on hydrophobic adhesion to embed randomly within the DM matrix, affording skeletal reinforcement (Figure 1c). Similarly, Teb@DC also exhibits lamellar stacking and cross‐linking microstructure (Figure 1b), but with a more pronounced interconnected network, indicating a higher cross‐linking density than Teb@DM, subsequent introduction of h‐BN into Teb@DC‐hBN yields an even higher nanosheet density (Figure 1d). The inverted vials demonstrate that every gel is self‐supporting and samples remain transparent in the absence of h‐BN, whereas a homogeneous milky white color develops after h‐BN incorporation, confirming its uniform distribution throughout the gel (Figure 1; Figure S2, Supporting Information).

Cryogenic scanning electron microscopy (cryo‐SEM) captured the native nano‐architecture of supramolecular sols in solvent environments. Both DM and DC supramolecular systems exhibit long‐range ordered fibrous structures, where interwoven fibers form lamellar networks, but DC strands pack more densely owing to citric acid's higher carboxyl density and stronger hydrogen bonding (Figure 1e,g). Boosting polyacid concentration amplifies more ordered and uniform lamellar structures with fibrous scaffolds showing dissociated fibrillar protrusions where DC lamellae straighten, betraying greater stiffness (Figure 1f,h). As for DM assembled with Teb, most localized regions display fungicide crystal‐embedded fibrous networks (Figure 1i), resulting in tighter cross‐linking with reduced pore size, which presumably ascribed to their synergistic interactions, including hydrophobic association between Teb and DMPSA as well as hydrogen bonding with polyacids. Upon dilution, Teb@DM sol reveals discrete fibrillar morphologies with broad fiber diameter distribution (70–470 nm, predominantly 100–200 nm) (Figure 1j), reflecting weakened aggregation behavior. In contrast, Teb@DC shows homogeneous Teb dispersion within the fibrous network (Figure 1k). It seems that Teb incorporation thickens lamellar scaffolds via hydrophobic stacking, thereby modifying interfacial properties. Furthermore, for h‐BN reinforced DM (DM‐hBN), nanosheets adhered to fiber scaffolds via hydrophobic interactions, with aggregates localized on fiber surfaces or pore junctions as displayed in Figure 1l. Comparative analysis of Teb@DM‐hBN and Teb@DC‐hBN reveals that h‐BN nanosheets embedded in fiber surfaces or acting as physical cross‐linkers, thus restricting network swelling and increasing cross‐linking density with more narrowed pore size distribution and widened fibers (Figure 1m–p). Besides, all sols remain macroscopically uniform and stable (insets in Figure 1e–p).

The particle size distributions of the aforementioned sol‐state samples are presented in Figure 1q–s. DM exhibits a normal distribution centered at 150–300 nm, whereas Teb@DM shows a narrower peak at 200–250 nm accompanied by a minor free‐Teb fraction at ≈75 nm (31 %), indicating the self‐assembly interactions between Teb and DM in aqueous solution promote size contraction of the DM matrix. Similarly, for the DC sol system, assemble with Teb results in a slight reduction in particle size from 201.27 ± 20.97 nm (DC) to 186.41 ± 5.85 nm (Teb@DC) (Figure 1r), demonstrating Teb's hydrophobicity‐driven contraction effect on the supramolecular assembly. In addition, the pH adjustment has a certain effect on the particle size, which is manifested as a slight decrease in particle size under acidic conditions compared with neutral conditions. In particular, compared the situations with CA and HCl are added to adjust the pH to 3, it is found that the addition of CA significantly reduces the particle size of DC and Teb@DC to 140.20 ± 4.27 and 115.99 ± 6.43 nm, which presumably attributed to CA preferentially protonates dimethylamine groups on DMPSA, occupying hydrogen bonding sites and enhancing hydrophobic association, thereby tightening the assembly. Under alkaline conditions, the DC and DM systems show different trends, which are manifested as a slight increase in the DC system and a slight decrease in the DM system. Such trends may be ascribed to deprotonation‐induced electrostatic repulsion and hydration layer expansion within DC system increasing the swelling of the assembly while fewer carboxyl groups in MA compared to CA, limiting charge density effects. The addition of h‐BN nanosheets leads to significant particle size increases of the supramolecular solution under all pH conditions (e.g., neutral pH: 760 nm) (Figure 1s). The fundamental reason is presumably attributed to the rigid lamellar structure of h‐BN, the surface chemical properties and its interaction with the supramolecular network jointly leading to the spatial hindrance effect, interface adsorption effect and pH‐dependent charge reconstruction. Finally, substitution of Teb with pyraclostrobin (Pyr), thiopyrad (Thi), fluopyram (Flp), or fluopyram (Flx) still yields nanoscale assemblies (Figure 1t), validating the platform as a universal nanocarrier for diverse fungicides.

### 2.2 Rheological Behavior

**Figure 2:**
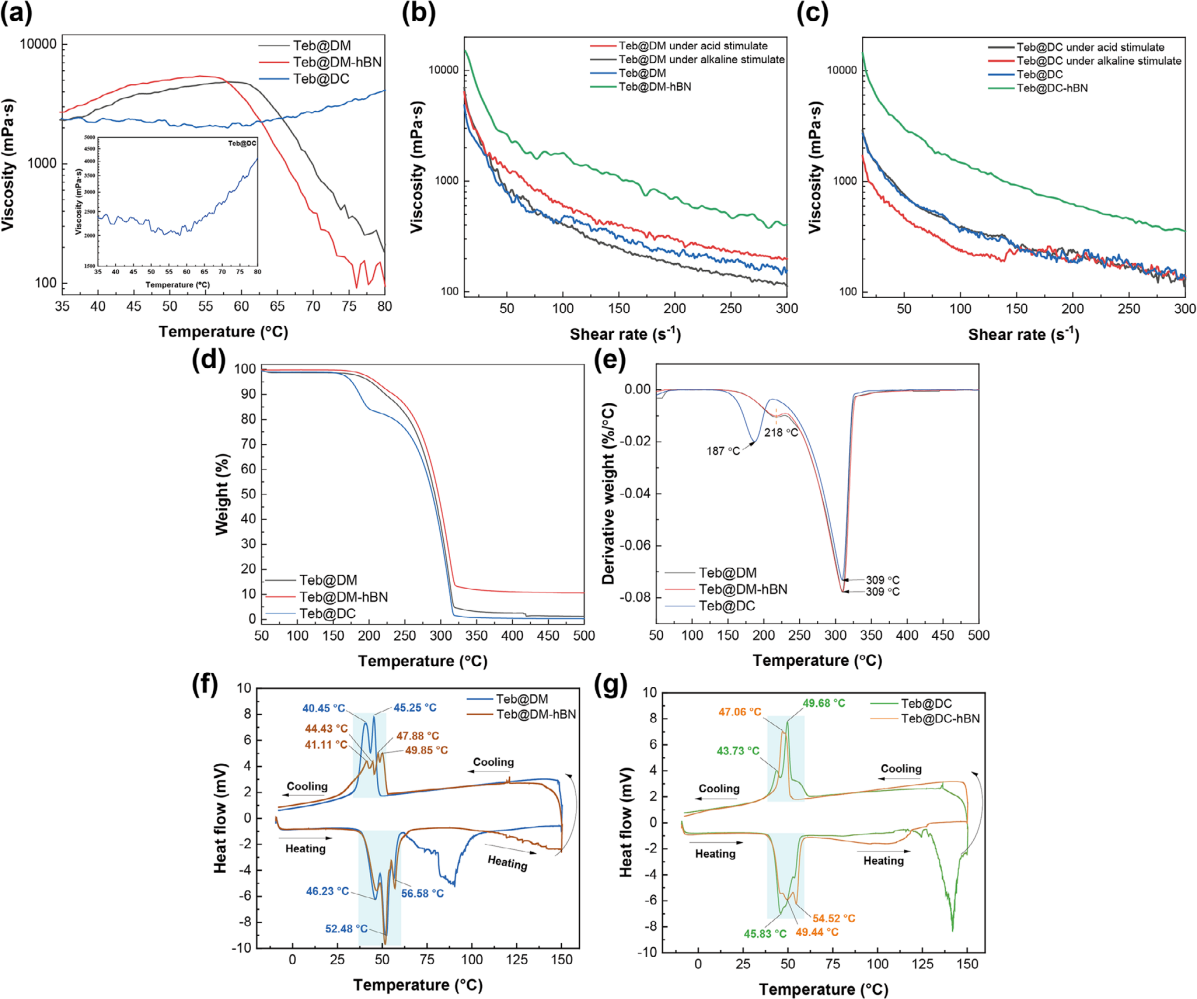
a) The viscosity variation curves of Teb@DM, Teb@DM‐hBN, and Teb@DC gels with temperature, b,c) the viscosity curves of Teb@DM and Teb@DC gels with shear rate increasing under different pH conditions, comparing the effect of h‐BN reinforcement on viscosity under shearing stress, d) TG and e) DTG curves of supramolecular lyophilized gels within the temperature range of 50–500 °C, f,g) DSC thermograms of supramolecular lyophilized gels within the temperature range of −10–150 °C.

In order to further explore the stimuli‐responsive properties of supramolecular gels, the viscosity variations with temperature and pH were characterized. As shown in **Figure** **2**a, for Teb@DM, moderate heating mobilises alkyl chains, creates weak hydrogen bonds that compensate for the rupture of stronger ones and drives the viscosity to a maximum of 4850 mPa·s at 58 °C, exhibiting a self‐reinforcing effect of physical cross‐linking. Further heating to 80 °C ruptures most hydrogen bonds, causing the viscosity to collapse to 185 mPa·s, suggesting a critical transition where hydrogen bond cleavage dominated over hydrophobic interactions at 58 °C. The addition of h‐BN accelerates heat transfer, making the Teb@DM‐hBN gel network respond earlier, and the maximum viscosity appears at 55 °C. Meanwhile, h‐BN's rigid surface provides additional binding sites, enhancing cross‐linking and increasing the peak viscosity to 5400 mPa·s at 55 °C. Intriguingly, the viscosity of Teb@DC remains almost stable, especially in the range of 35–58 °C, as ionic cross‐linking from CA's tricarboxylate groups dominated, and then the viscosity shows an upward trend after 58 °C, reaching ≈4100 mPa·s at 80 °C. This may be due to the gradual deprotonation of the tricarboxylic acid (CA) during the heating process, which significantly alters the network's charge state, triggering hydrophobic reorganization with alkyl chains to form thermally stable microdomains, thereby achieving high‐temperature viscosity recovery. The thermosensitive viscosity of supramolecular gel arises from a triple interplay of hydrogen bonding, hydrophobic interactions, and ionic cross‐linking. Through leveraging h‐BN's thermal regulation or ion‐to‐hydrophobic switching of polycarboxylic acids, intelligent thermal‐responsive systems based on supramolecular gels can be designed for precision agrochemical delivery.

In addition, both Teb@DM and Teb@DC supramolecular gels exhibit shear‐thinning behavior (Figure 2b,c). This property facilitates spray applications by reducing viscosity under shear (e.g., during nozzle atomization) while maintaining high viscosity after deposition. The viscosity of Teb@DM increases under acid stimulation due to protonation of dimethylamine groups on DMPSA, enhancing hydrophobic association and hydrogen bonding, whereas the viscosity decreases under alkali stimulation due to deprotonation of carboxylic acid groups, increasing electrostatic repulsion and hydration layer thickness. In contrast, the viscosity of Teb@DC shows minimal viscosity change under acid stimulation, as CA pre‐occupies protonation sites, limiting further network contraction, while the viscosity of Teb@DC under alkali stimulation also shows a downward trend owing to the carboxyl groups dissociating. The combination of shear‐thinning and pH‐tunable viscoelasticity yields environment‐adaptive fungicide delivery that can be matched to field conditions.

Besides, at the initial low shear rate, the viscosities of Teb@DM and Teb@DC show zero‐shear viscosities of 4907 and 2708 mPa·s, but these values jump to 14903 mPa·s (×3.64) and 14497 mPa·s (×5.35) upon incorporation of h‐BN nanosheets, giving a direct, quantitative index of markedly improved mechanical stability and structural integrity of the gel network. Importantly, even at a high shear rate of 300 s^−1^, the viscosities of the h‐BN reinforced gels remain significantly higher than their neat counterparts, indicating robust network strength under stress. The likely reasons for this reinforcement may be attributed to the h‐BN nanosheets acting as multifunctional nano‐fillers within the supramolecular gel matrix. They can serve as physical cross‐linking points, reinforcing the 3D network skeleton; form strong interactions (e.g., hydrophobic, *π*–*π* stacking) with the organic components of the gel, restricting chain mobility; and narrow the pore distribution, leading to a denser and more rigid structure.

### 2.3 Thermodynamical Behavior

Thermogravimetric analysis (TGA) was carried out in an N_2_ atmosphere to investigate the decomposition behavior of supramolecular lyophilized gels as the temperature increased. The nano‐assemble with Teb effects mainly reflect that carbon chain decomposition shifts to 309°C in comparison with DM and DC, while hydrogen bond cleavage temperatures remain almost unchanged (Figure 2d,e; Figure S6a,b, Supporting Information). Intriguingly, Teb@DM and Teb@DC show similar carbon chain decomposition temperatures (309 °C), suggesting Teb's hydrophobic interactions modulate thermal stability of the carbon backbone. And weight loss reduction at hydrogen bond cleavage temperatures indicates Teb enhancing cross‐linking density in the supramolecular network. The incorporation of h‐BN does not alter the thermal decomposition temperatures of supramolecular components. When heated to 500 °C, it can be inferred from the weight loss percentage that the mass fraction of h‐BN within the Teb@DM‐hBN system is calculated as ≈8.13% based on residual weight.

Differential scanning calorimetry (DSC) was employed to elucidate the phase transition behavior of the gels, as shown in Figure 2f,g and Figure S6c (Supporting Information). Both Teb@DM and Teb@DC exhibit reversible heating‐cooling cycles between −10 and 150 °C, confirming the thermally recyclable nature of the fungicide‐assembled supramolecular gels. Teb@DM forms three endothermic peaks between 37.49–61.71 °C, implying triple interaction forces. The cooling exothermic transition of Teb@DM becomes two peaks at 45.25 and 40.45 °C, respectively. In contrast, the hydrogen bond network in DC supramolecular gel may be more homogeneous, resulting in the single merged endothermic peak appearing at 61.13 °C, while Teb@DC exhibits one dominant peak at 45.83 °C with additional two weak shoulder peaks, indicating slight heterogeneity induced by Teb, and the phase transition temperatures during heating and cooling are closer with minimal hysteresis, indicating a stable phase transition process. The addition of h‐BN has negligible change on the endothermic peak during the heating process of Teb@DM‐hBN, whereas causing the peak splitting in exothermic regime during the cooling process revealing the multi‐step reassembly, as well as significantly reducing the heat flow intensity. The addition of h‐BN indicates more kinetically stabilized phase transitions and reassembly of supramolecular gels.

### 2.4 Protonation Effect Analysis

**Figure 3:**
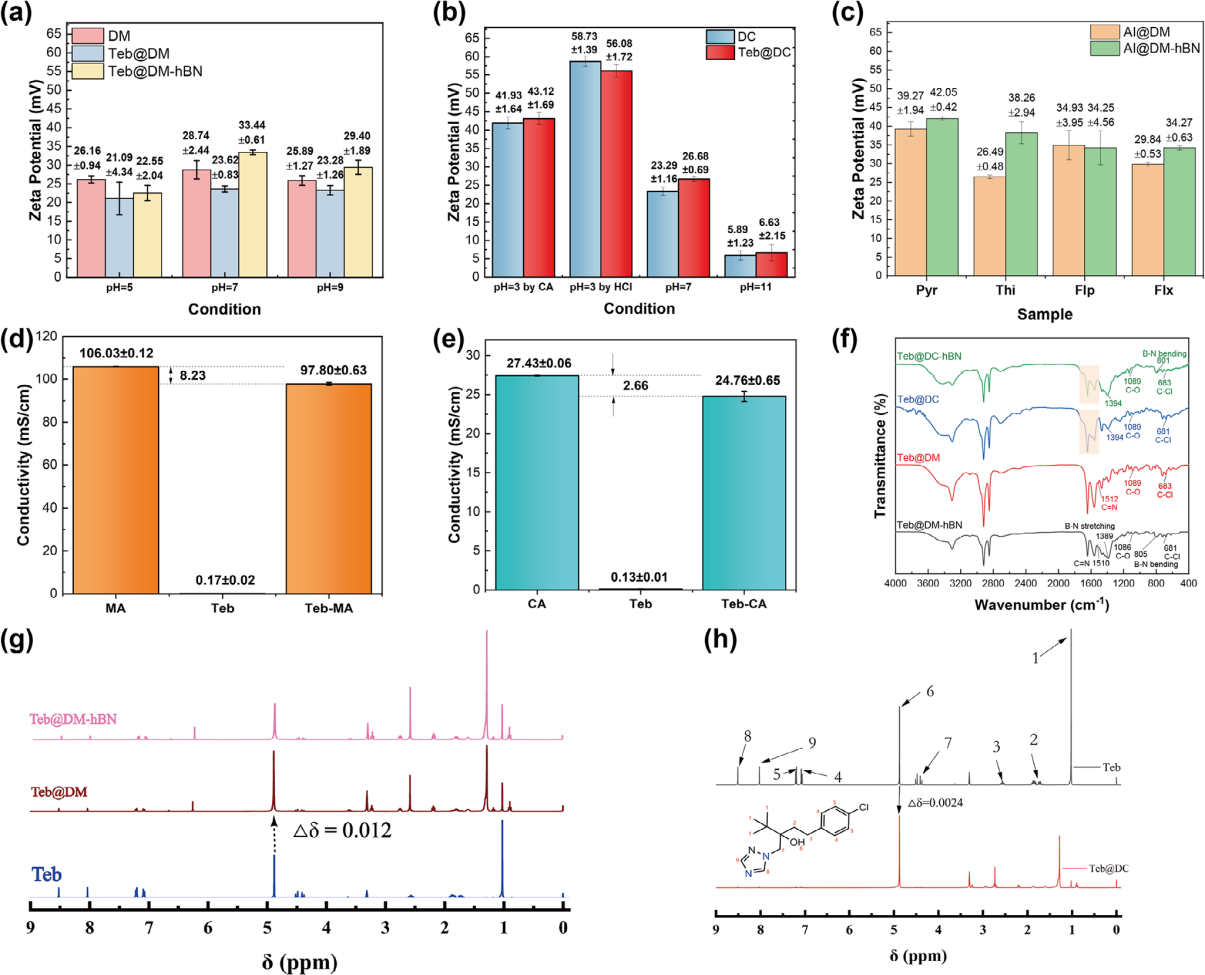
a) Zeta potential values of sol‐state DM, Teb@DM, Teb@DM‐hBN under different pH conditions, b) zeta potential values of DC and Teb@DC under different pH conditions, c) zeta potential values of other fungicides assembled within DM and DM‐hBN, d) the conductivity values of MA, Teb, and Teb‐MA, e) the conductivity values of CA, Teb, and Teb‐CA, f) FTIR spectra of Teb@DM, Teb@DC, and corresponding h‐BN reinforced Teb@DM and Teb@DC in the range of 4000–400 cm^−1^, g) ^1^H NMR spectra of Teb, Teb@DM, Teb@DM‐hBN, h) ^1^H NMR spectra of Teb and Teb@DC.

The charge interactions within sol‐state fungicide‐assembled supramolecular systems were investigated through zeta potential measurements, as shown in **Figure** **3**a–c. DM and DC both exhibit positive surface charges. DM shows minimal potential variation across pH, attributed to MA's limited buffering capacity with pKa_1_ = 1.9 and pKa_2_ = 6.1. However, DC displays zeta potential surges under acidic condition due to protonation of CA's tertiary carboxyl groups, eliminating negative charges and amplifying net positive charge, while the zeta potential drops under alkaline condition as CA undergoes complete deprotonation, generating negative charges that partially neutralize DMPSA's positive groups. Such comparison may attribute to CA's broader pKa range (3.1, 4.8, and 6.4) enables multi‐stage proton transfer. After DM assembled with Teb, the zeta potential decreases slightly. On the contrary, the zeta potential of DM assembled with other fungicides increase, such as Pyr@DM, Flp@DM, and Flx@DM. This may be attributed to the fact that Teb's weakly electronegative chlorine atom partially neutralizes DMPSA's positive charge, while Pyr/Flp/Flx all have benzene rings or fluorine atoms, which may expose the charged groups more through π‐cation interaction or hydrophobic collapse. The zeta potential of DC assembled with Teb in a neutral solution also increases slightly, which may be due to Teb strengthening ion‐pair formation between CA and DMPSA, while hydrophobic compression of the double layer reduces carboxyl protonation, amplifying net positive charge and reflecting the local charge recombination. The disparity reflects matching compatibility between fungicide polarity and supramolecular surface charge. By comparison, it is found that the addition of h‐BN uniformly increases the zeta potential of the fungicide‐assembled DM via two synergistic mechanisms, including electrostatic double‐layer compression and dielectric confinement. h‐BN's negative surface charge compresses the diffuse layer around positively charged DM and the rigid sheet hinders the diffusion of counterions, as well as its high dielectric constant restricts ion diffusion, effectively amplifying the surface potential through electrostatic shielding. This phenomenon also provides a strategy for charge regulation in the design of environmentally responsive fungicide formulations.

The interaction between Teb and MA or CA was investigated via conductivity measurements. As shown in Figure 3d,e, the conductivity values of MA and CA are 106.03 ± 0.12 and 27.43 ± 0.06 mS cm^−1^, respectively. The conductivity of Teb alone is extremely low, only 0.12–0.17 mS cm^−1^, consistent with its non‐ionic nature. However, when MA or CA with Teb forms a mixed system, the conductivity values of the system drop to 97.80 ± 0.63 and 24.76 ± 0.63 mS cm^−1^, respectively. The essence of the reduction in conductivity is that Teb's hydrophobic groups (benzene/triazole rings) associate with acid anions via hydrogen bonding, π‐π interactions, hydrophobic aggregation shielding ions from solvation, creating an ion‐masking effect, impeding charge migration. In particular, CA's triple carboxyls offer more hydrogen bonding sites, leading to greater conductivity reduction (9.70%). MA's cis‐double bond may enable Teb to bridge two carboxyls, introducing steric hindrance that disrupts ion hydration, leading to the conductivity decrease by 7.76%. Combined conductivity data analysis with the zeta potential analysis, Teb may change the local charge environment based on various acids.

### 2.5 Chemical Interaction Analysis

Fourier‐transform infrared spectroscopy (FTIR) was utilized to probe the supramolecular interactions, as depicted in Figure 3f and Figure S7a,b (Supporting Information). In Teb, imidazole C═N sits at 1510 cm^−1^ and C─Cl at 680 cm^−1^, upon assembly into DM the C═N band red‐shifts to 1512 cm^−1^, indicating MA‐protonation of the triazole ring, while the C─O band at 1089 cm^−1^ stays intact. Teb@DM‐hBN additionally shows B‐N stretch (1389 cm^−1^) and bend (805 cm^−1^), confirming h‐BN incorporation. ^1^H NMR (Figure 3g,h; Figure S7c,d, Supporting Information) reveals that the Teb hydroxyl proton moves 0.012 ppm in Teb@DM but only 0.0024 ppm in Teb@DC. This shows that different polycarboxylic acid supramolecular systems have varying hydrogen bonding interactions with active ingredients. Besides, XRD (Figure S8b, Supporting Information) shows a decreased 2θ angle, corresponding to an increase in the interlayer spacing, attributed to the effective intercalation of Teb molecules into the interlamellar galleries of h‐BN. This intercalation is driven by *π*–*π* stacking interactions between the benzene rings of the Teb molecule and the boron‐nitrogen hexagonal rings in the h‐BN structure.

Besides, Teb, Teb‐DMPSA, Teb‐MA, and Teb‐CA were also measured by XPS to identify the hydrogen bonding sites of MA and CA with Teb, as depicted in Figure S9c_1_–c_4_,d_1_–d_4_ (Supporting Information). In the XPS Cl spectra (Figure S9c_1_–c_4_, Supporting Information), the peaks at 202.3 and 200.6 eV (with an area ratio of 1:2) are assigned to the intrinsic Cl features. The peak at 197.6 eV indicates free Cl^−^ ions in Teb. In Teb‐MA and Teb‐CA, the 197.6 eV peak disappears, leaving only the intrinsic 200.6 eV peak, showing that the carboxyl groups within MA and CA alters the Cl electron environment through strong electron‐withdrawing effects, bringing the Cl^−^ binding energy closer to that of C─Cl and merging it with the main peak. In the XPS N 1s spectra (Figure S9d_1_–d_4_, Supporting Information), the main peak of Teb's N 1s appears at 399.65 eV (68.87%) corresponded to N in the triazole ring. The peak at 401.59 eV (31.13%) indicated some N atoms formed hydrogen bonds. The DMPSA addition merely changes Teb's intrinsic N 1s peak, implying no significant interaction between them. However, after adding MA, a new peak at 402.29 eV (protonated N) appears in Teb‐MA. Similar results are shown in Teb‐DC. This confirms that MA and CA form hydrogen bonds with N in Teb's triazole ring, protonating Teb.

### 2.6 Loading Capacity and Sustained Release Performance

**Figure 4:**
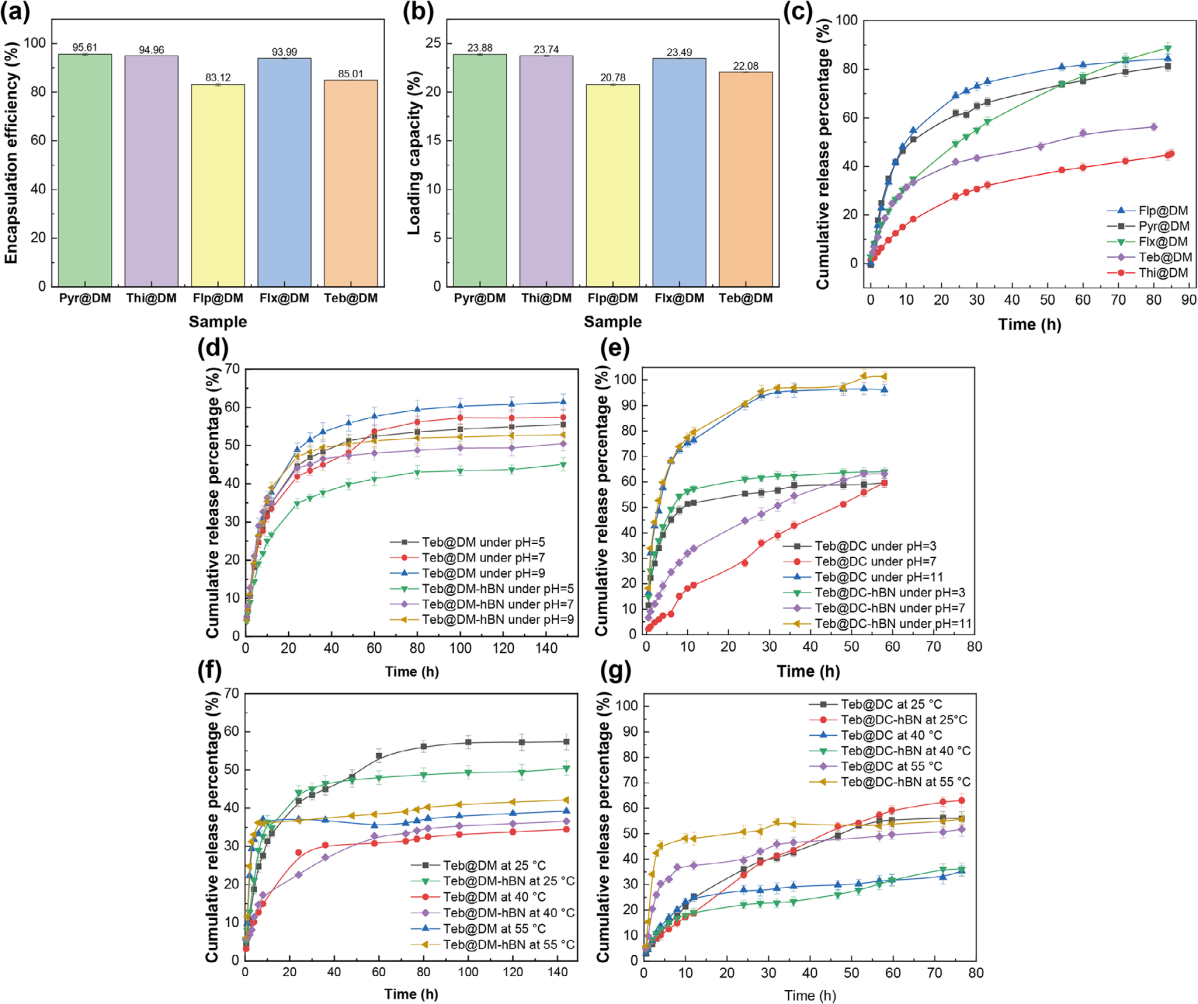
a) Encapsulation efficiency file and b) loading capacity file of DM assembled with various active ingredients, c) cumulative release profile of various active ingredients from DM supramolecular gel, pH‐responsive cumulative Teb release profiles of d) Teb‐assembled DM and Teb‐assembled DM‐hBN supramolecular gel and e) Teb‐assembled DC and Teb‐ assembled DC‐hBN under different environmental pH conditions, thermo‐responsive cumulative Teb release profiles of f) Teb‐assembled DM and Teb‐assembled DM‐hBN and g) Teb‐assembled DC and Teb‐assembled DC‐hBN under different environmental temperature conditions.

The encapsulation efficiency and loading capacity of supramolecular gels assembled with various fungicides were investigated. As can be seen from **Figure** **4**a,b, the encapsulation efficiencies of the five fungicides assembled by DM are all above 80%, and correspondingly, the loading capacity are all between 20% and 25%, indicating that the supramolecular gels have good loading performance. Among different specific fungicides, Flp@DM and Teb@DM exhibit relative lower encapsulation efficiency (83.12 ± 0.48% and 85.01 ± 0.13%, respectively) and similar trends are also reflected in the loading capacity with the values of 20.78 ± 0.10% and 22.08 ± 0.02%, respectively. The possible reason for such trend is the larger molecular structure or poorer solubility. Therefore, such supramolecular gels has a certain universality for active ingredients loading.

Based on the sustained release profile of five fungicides assembled by supramolecular gels in Figure 4c, it is shown that there are significant differences in the sustained release behavior of the five fungicides from DM supramolecular gels. Pyr, Flp, and Flx release faster from DM gels than the other three cases, where most of the fungicides release (>81%) within 80 h, while Thi@DM exhibits the lowest cumulative sustained release percentage with the value of 45.26% at 80 h attributed to the multiple hydrogen bond networks and hydrophobic interactions formed by its thioether group (‐S‐) with MA within DM, limiting the diffusion rate of active ingredient molecule. This shows that the sustained release performance of active ingredient is closely related to molecular structure and supramolecular assembly. Obviously, Teb@DM forms a relative moderate release profile. The possible sustained release mechanism is that when the driving force of the solvent molecules penetrating the gel network and the elastic contraction force of the cross‐linked network reach a dynamic equilibrium, the swelling stops. At this time, the active ingredient release mainly depends on the diffusion process inside the gel network, and the release rate naturally slows down.

### 2.7 pH‐Responsive Controlled Release Behavior

Figure 4d compares pH‐triggered Teb release from Teb@DM and Teb@DM‐hBN. After 80 h the profiles plateau, reflecting dialysis equilibrium governed by van der Waals and hydrogen bond interactions between the hydrophobic fungicides and the supramolecular carrier. Both Teb@DM and Teb@DM‐hBN exhibit identical pH‐responsive release patterns. At 144 h, the cumulative release rate drops from basic to acidic conditions. This is mainly due to the supramolecular DM's pH responsiveness. Under weakly acidic solutions, reverse ionization of maleic acid lowers free protons, prompting extra hydrogen bonds between carboxyls and DMPSA that tighten the network and suppress Teb release. Conversely, hydroxide ions at high pH disrupt these bonds and accelerate release, mirroring the pH‐rheology of DM. Furthermore, h‐BN lowers the overall release rate through hydrophobic affinity to DM's long carbon chains and *π*–*π* contacts with Teb, which jointly hinder diffusion.

For comparison, pH‐dependent release of DC was studied (Figure 4e). The highest cumulative release rate occurs at pH 11 where OH^−^ ions disrupt hydrogen bonds and destabilize the network, causing rapid Teb release. This matches the low zeta potential observed under basic conditions. In contrast, at pH 3 extensive protonation renders the carrier fluid‐like, so the rate exceeds that at gel‐like pH 7. Teb@DC and Teb@DC‐hBN exhibit almost identical release profiles, indicating that h‐BN scarcely affects the DC system. Comprehensively, DM forms a flexible hydrophobic matrix, allowing h‐BN to insert among alkyl chains, enhance hydrophobic association and slow release, whereas DC builds a rigid ionic network through citric acid tertiary carboxyls, so high‐energy ion pairs are hardly perturbed and Teb continues to diffuse through the existing ion channels.

### 2.8 Thermo‐Responsive Controlled Release Behavior

Figure 4f shows that Teb release from both DM and DM‐hBN supramolecular gels exhibits an unusual deceleration as temperature rises. Specifically, cumulative release peaks at 25 °C, drops markedly at 40 °C, and recovers somewhat at 55 °C, creating two clear inflection points. The two inflection points (40 and 55 °C) suggest the existence of two activation energy transition points. The fundamental reason lies in the differentiated regulation of temperature on the dual forces of the supramolecular network and the topological constraint effect of h‐BN. Originally, hydrogen bonds dominate and the network is loose at 25 °C, then, hydrophobic association is enhanced to form a dense structure at 40 °C, intriguingly, a large number of hydrogen bonds may be broken and the network becomes loose at 55 °C. The addition of h‐BN is equivalent to adding a skeletal scaffold to the network, weakening the structural fluctuations caused by temperature.

For comparison, Figure 4g presents the temperature‐dependent release of Teb@DC. At 25 °C the profile is almost linear, dominated by simple diffusion; but at 40 and 55 °C the rate declines, though by distinct mechanisms. At 40 °C tight hydrophobic association continuously locks the active ingredient, whereas at 55 °C an initial burst is followed by slower release ascribed to the network reorganized to form a diffusion barrier and breaking the hydrogen bonds. Hydrogen bond scission momentarily opens channels, yet subsequent hydrophobic reorganisation rebuilds a diffusion barrier. Beside highlighting the triple interplay of ionic, hydrophobic and hydrogen forces, this temperature‐tunable behavior offers a molecular switch for environmentally adaptive pesticide design. This may be related to the differential effects of temperature on the triple forces of DC.

### 2.9 Wetting and Adhesion Characteristics on Foliar Surfaces

**Figure 5:**
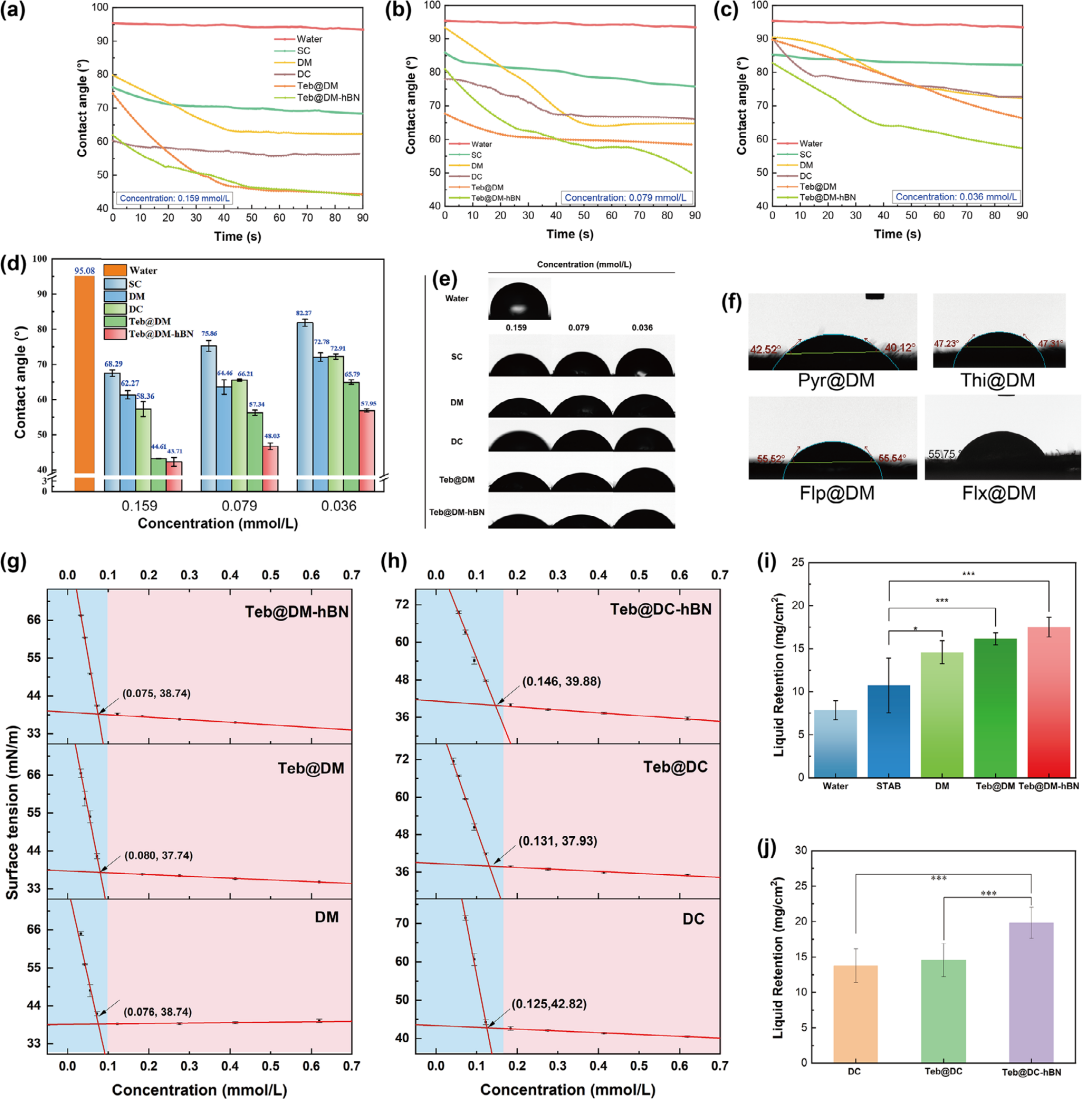
a–c) The dynamic contact angles of supramolecules at different concentrations (0.159, 0.079, and 0.036 mmol L^−1^) while using water and SC as controls on cucumber leaves, d,e) contact angles histogram and snapshots of various samples at different concentrations at the measuring time of 90 s, f) contact angles of various fungicides‐assembled DMs on cucumber leaves. The variations of surface tension of g) DM, Teb@DM, Teb@DM‐hBN, and h) DC, Teb@DC, Teb@DC‐hBN with concentration increase, the liquid retention capabilities of i) DM, Teb@DM, Teb@DM‐hBN, and j) DC, Teb@DC, Teb@DC‐hBN, using water and STAB as controls (***: *p *≤ 0.001, **: *p* ≤ 0.01, *: *p* ≤ 0.05).

To evaluate the wetting ability of supramolecular systems, hydrophobic cucumber leaves were used as a model. As shown in **Figure** **5**a–c, DM, DC, Teb@DM, and Teb@DM‐hBN all give dynamic contact angles far below commercial Teb suspension concentrate (SC) formulation, which stems from DMPSA's innate surface activity and hydrogen bonding with leaf wax, promoting spread. The dynamic contact angles of Teb@DM and Teb@DM‐hBN continuously decline, so the droplet self‐spreads and roll‐off loss is cut. Even at lower concentrations (Figure 5c), the contact angles of DM and DC still decrease, proving good dilution‐spread that allows dose reduction. Teb further lowers the angle by tightening the fibrous network via hydrogen bonds. The addition of h‐BN even more reduces the contact angle, likely due to its inherent hydrophobicity and forming capillary channels at the interface, further facilitating the infiltration of the solution and enhancing adhesion to hydrophobic leaf surfaces. Various fungicides‐assembled DMs on cucumber leaves also exhibit significantly reduced contact angle and smaller than the value of DM (Figure 5f), confirming that hydrogen bond and hydrophobic cooperation between any fungicide and DM universally improves wetting. Herein, the boron nitride‐reinforced supramolecular gels exhibit more uniform distribution and excellent spreading on the leaf surface.

Surface tension titrations (Figure 5g) reveal that DM, Teb@DM, and Teb@DM‐hBN share critical micelle concentration (CMC) values of 0.075–0.080 mmol L^−1^ and surface tensions near 38 mN m^−1^, enabling efficient wetting. In sharp contrast, DC has a CMC of 0.125 mmol L^−1^, 55.42% higher than the case of DM (Figure 5h) because citric acid's extra carboxyls form intramolecular hydrogen bonds that self‐lock the molecules and curb micellization. Adding h‐BN or Teb to DC further raises the CMC. When h‐BN is added to DC, the B‐OH groups at its edges compete with the carboxyl groups of citric acid for proton acceptors, which is like a third party (h‐BN) suddenly entering the dance floor and snatching the original dance partner (proton), disrupting the original ion pairing rhythm. The chlorobenzene ring of Teb acts like a wedge, inserting between the carboxyl groups of citric acid and hindering the formation of an effective ionic cross‐linking network. However, in the DM system, maleic acid has a compact structure, and the interference of h‐BN and Teb is like sprinkling water on a stone, having limited impact. An increase in CMC means a decrease in micelle stability, but it makes the system more responsive to pH‐triggered dissociation and rapid release of pesticides. In contrast, the DM system is more suitable for long‐term controlled release.

Hydrophobic cucumber leaves were used to evaluate adhesion (Figure 5i). STAB solution, containing a surfactant with the same long chain as DMPSA, retains 10.73 mg cm^−2^, 36.86% more than water, owing to reduced surface energy and enlarged contact area. However, DM and Teb@DM further raise retention by ≈35.97% and ≈50.61%, respectively, because maleic acid provides extra hydrogen bonds and nano‐scale effects boost leaf affinity. The addition of h‐BN increases deposition of Teb@DM through additional hydrophobic interaction with the cuticle. Overall, adhesion levels of DM and DC series are comparable, confirming DMPSA as the main adhesive component while the acid type plays a minor role. Thus, Teb@DM‐hBN and Teb@DC‐hBN effectively anchor active ingredients on leaves, cut droplet loss and improve pesticide utilization.

**Figure 6:**
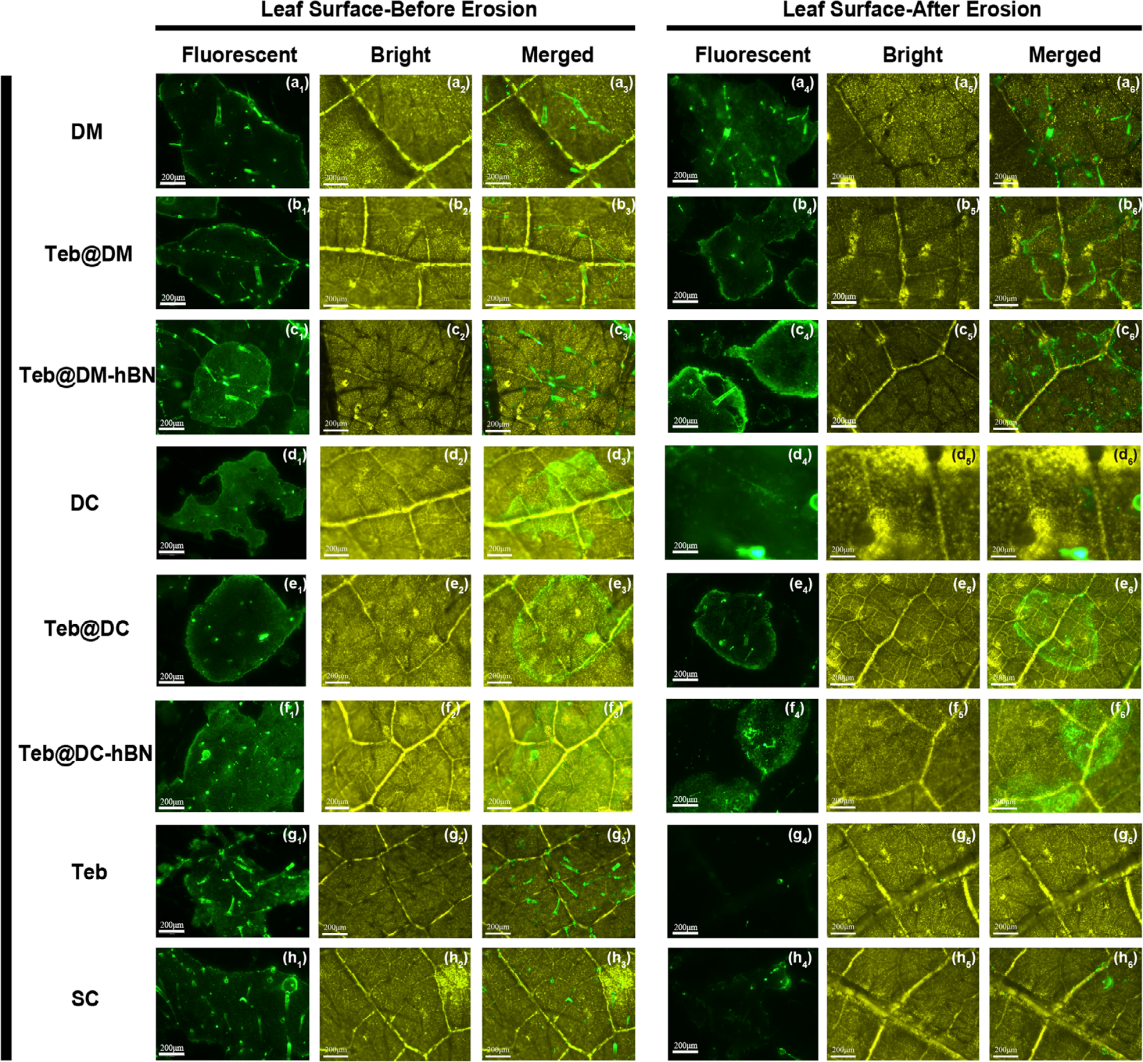
Inverted fluorescence images of cucumber leaf surfaces rinsing with DM a_1_–a_3_), Teb@DM b_1_–b_3_), Teb@DM‐hBN c_1_‐c_3_), DC d_1_–d_3_), Teb@DC e_1_–e_3_), Teb@DC‐hBN f_1_–f_3_), Teb g_1_–g_3_), and SC h_1_–h_3_), and the corresponding captured images after erosion.

Inverted fluorescence microscope was used to observe the distribution of FITC‐labelled formulations on cucumber leaves before and after simulated rainfall, evaluating their adhesion and wash resistance on hydrophobic surfaces (**Figure** **6**; Figure S12, Supporting Information). Before rinsing, all samples are observed distinct fluorescence on the leave surfaces, nervures, and trichomes. However, free Teb signal almost vanishes after erosion, while commercial SC retains only weak fluorescence, indicating poor adhesion. In contrast, the DM, Teb@DM, and Teb@DM‐hBN supramolecules maintain high intensity, demonstrating that supramolecular gels markedly improve rain‐fastness. Likewise, DC, Teb@DC, and Teb@DC‐hBN supramolecules elucidate the similar enhancement trends against erosion. Cuticular wax (long‐chain fatty acids, alcohols, aldehydes) provides hydrogen bonding sites for the gels, while positive zeta potential of the supramolecules enables electrostatic attraction to negatively charged leaf groups, enhancing deposition. Intriguingly, the h‐BN reinforced Teb@DM and Teb@DC exhibit the strengthen fluorescence intensity compared to their neat counterparts, attributed to boron nitride's strong leaf affinity, further boosting adhesion.

### 2.10 Supramolecules and Soil Interaction Analysis

**Figure 7:**
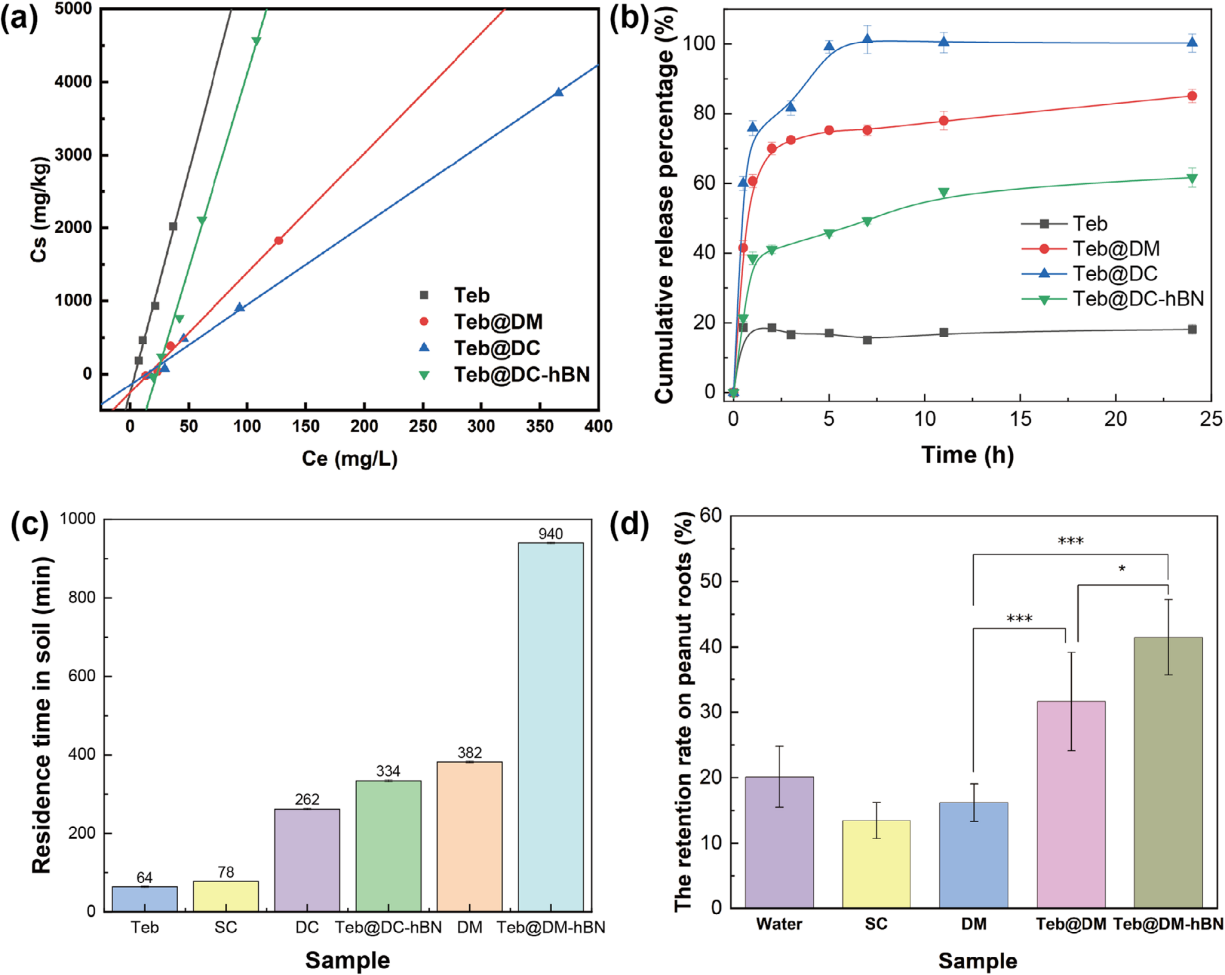
a) The adsorption isotherms of sandy loam for Teb, Teb@DM, Teb@DC, and Teb@DC‐hBN, b) the cumulative release percentages of free Teb and Teb‐assembled supramolecules from sandy loam soils within 24 h, c) the residence time of supramolecules through soil column during leaching, using free Teb and commercial SC as controls, d) the retention capacity on peanut roots treated by water, SC, DM, Teb@DM, Teb@DM‐hBN (***: *p* ≤ 0.001, **: *p* ≤ 0.01, *: *p* ≤ 0.05).

The adsorption isotherms of sandy loam soil for Teb, Teb@DM, Teb@DC, and Teb@DC‐hBN are shown in **Figure** **7**a. The isothermal adsorption data of the four samples were fitted using the Freundlich and linear models, and the relevant parameters are presented in **Table** **1**. The isothermal adsorption data of the four samples on sandy loam soil displays a weak fit with the Freundlich model (*R* ^2^ < 0.97) but a better fit with the linear model (*R* ^2^ > 0.99). This indicates that the adsorption of Teb and supramolecular gels on the soil surface is closer to monolayer adsorption or occurs on a relatively uniform energy surface, suggesting that the adsorption process is predominantly physical with weak interactions between adsorbate molecules. The K_d_ value, which indicates the soil's adsorption capacity for a substance, reveals that the adsorption performance of the four samples on sandy loam soil follows the order: Teb > Teb@DC‐hBN > Teb@DC > Teb@DM. The adsorption of free Teb onto the soil is the strongest. This can be evidenced by the steepest slope of the adsorption isotherm and the largest K_d_ value. Such phenomenon is attributed to the high degree of freedom of the Teb molecules, which can readily penetrate into the nanopores of soil organic matter. However, the supramolecular carriers act as a kind of coating for Teb. The hydrophilic outer shells of DM or DC supramolecules impede the interaction between Teb and hydrophobic organic matter. This can be verified by the decreased slope of the adsorption isotherm and the significant decline in the K_d_ values of Teb@DM and Teb@DC. The soil adsorption capacity of the Teb@DM supramolecule is slightly higher than the case of Teb@DC, with a marginally higher K_d_ value and a slightly steeper slope. This difference stems from the variations in charge characteristics, spatial configurations, and competition for soil interaction sites among the two supramolecular structures. The presence of boron nitride enhances the adsorption of the Teb@DC supramolecule onto the soil. The lamellar structure of h‐BN disrupts the integrity of the supramolecular outer shell. It is analogous to creating a breach in a protective suit, thereby exposing a portion of Teb. Although the supramolecular carriers reduce the initial adsorption of Teb, h‐BN counteracts the low adsorption drawback of the supramolecular carriers via the interfacial effect. This implies that in regions with a high risk of leaching, the selection of Teb@DC‐hBN, which exhibits moderate adsorption, is advantageous for minimizing groundwater contamination. This finding provides a theoretical foundation for the precise design of soil‐tailored control agents against soil‐borne fungal diseases.

**Table 1:** Sandy loam adsorption isotherm data for four samples with fitting parameters by the Freundlich model and the linear model.

Soil	Sample	Freundlich model			Linear model			Adsorption level	△G
		K_f_‐ads	1/n	*R* ^2^	K_d_	*R* ^2^	K_oc_		
Sandy loam	Teb	13.74	1.391	0.976	60.59	**0.990**	605890	Highly adsorbable in soil	−33.00
	Teb@DM	0.141	2.065	0.766	16.41	**0.993**	164150		−29.77
	Teb@DC	1.292	1.391	0.889	10.97	**0.997**	109740		−28.77
	Teb@DC‐hBN	0.239	2.143	0.973	53.26	**0.995**	532590		−32.68

The soil adsorption free energy (ΔG) is a key parameter for characterizing the adsorption properties between soil and samples and is crucial for evaluating the adsorption extent and mechanism of nanopesticides in soil. As listed in Table 1, the experimental data show that the ΔG values for Teb, Teb@DM, Teb@DC, and Teb@DC‐hBN in sandy loam soil are all negative and have absolute values below 40 kJ mol^−1^. This confirms that the adsorption mechanism is primarily physical and exothermic, indicating that reducing the ambient temperature can enhance the adsorption efficiency of supramolecular gels assembled with Teb in soil.

The desorption capacity of pesticides reflects their release rate and potential mobility in soil, and characterizing this property supports the development of controlled‐release pesticides. Herein, the sustained‐release performance of Teb, Teb@DM, Teb@DC, and Teb@DC‐hBN from sandy loam soil was evaluated (Figure 7b). The cumulative release curves display that Teb@DC exhibits rapid initial release (near 100% within 5 h), indicating weak soil interaction and quick desorption. Teb@DM and Teb@DC‐hBN show slower and sustained release behavior, reaching ≈85% and 60% cumulative release after 24 h, likely due to stable adsorption with the soil. In contrast, Teb has only ≈15% cumulative release, indicating strong soil adsorption and poor desorption. The cumulative release order from soil within 24 h is Teb@DC > Teb@DM > Teb@DC‐hBN > Teb, exactly with the opposite trend against the soil adsorption performance. This highlights the significant impact of supramolecular carrier's type on soil release kinetics with the behavior of DC carriers favoring rapid release, while DM carriers enabling slow and sustained release. Notably, the incorporation of boron nitride enhances carrier‐soil affinity, further promoting sustained release. These findings provide a basis for selecting supramolecular carriers in soil applications, where DC and DM for rapid action and h‐BN‐reinforced supramolecular carriers for prolonged efficacy.

The retention time of pesticides in soil is critical for their efficacy in controlling soil‐borne diseases. Aiming this, a soil column leaching experiment was used to assess the retention capacity of supramolecules, measured by the residence time taken for the last drop to fall from the soil column (Figure 7c). The comparison exhibits that DC and DM supramolecules possess residence time of 262 and 382 min, respectively, significantly longer than Teb's 64 min and SC's 78 min, indicating better retention capacity in soil. Further analysis reveals that h‐BN incorporated into the supramolecules prolongs their retention time with Teb@DC‐hBN and Teb@DM‐hBN achieving residence time of 334 and 940 min respectively, outperforming DC and DM. This demonstrates that h‐BN enhances supramolecules and soil interactions, thereby improving retention capacity. Although Teb exhibits high adsorption capacity in soil as aforementioned, it demonstrates quickly flow and short retention time through soil. Hence, the properties and composition of the supramolecules significantly impact their retention in soil. Notably, adding h‐BN can markedly enhance soil retention capacity of fungicides against soil‐borne fungal diseases.

Visually, white h‐BN within supramolecules remains around the top 10 cm soil depth during leaching experiments (Figure S13, Supporting Information), indicating it can effectively retain active substances in the soil surface. This makes it highly efficient for controlling soil‐borne diseases and prevents leachable fungicides from contaminating groundwater.

### 2.11 The Interaction Analysis between Supramolecules and Plant Roots

The adhesive and retention capability of supramolecules on plant roots was evaluated by peanut root immersion tests. The results in Figure 7d illustrate that Teb@DM‐hBN holds the highest retention rate at 41.45% on peanut roots, outperforming others. Teb@DM has a 31.66% retention rate, higher than the value of DM (16.20%) and SC (13.48%). DM's high surface activity likely caused its low root adhesion. Teb@DM‐hBN's strong adhesion to peanut roots makes it more effective for controlling soil‐borne diseases. This aligns with previous findings that adding h‐BN boosts the adhesion of fungicide‐assembled supramolecules on plant roots, enhancing fungicide efficiency and better protecting peanut growth.

### 2.12 Antifungal Activity In Vitro

The antifungal activity of Teb@DM, Teb@DM‐hBN, Teb@DC, and Teb@DC‐hBN against soil‐borne fungal diseases (*Sclerotium rolfsii*) was evaluated under the same Teb concentrations, with results shown in Figure S14 (Supporting Information). The data indicate that Teb, Teb@DM, and Teb@DM‐hBN all have certain inhibitory effects on *Sclerotium rolfsii*, with the fungicidal activity primarily stemming from Teb. However, the antifungal activity between free Teb and Teb‐assembled supramolecules exhibit different trends over time. As the number of days increased, the mycelium expands outward from the center of the colony, leading to a gradual decrease in the inhibition rate. On the sixth day, the inhibition rates of Teb, Teb@DM, and Teb@DM‐hBN are 64.02%, 70.35%, and 80.27%, respectively. The higher inhibition rates of Teb@DM and Teb@DM‐hBN compared to free Teb suggest that the supramolecular system slow the release of Teb, enhancing its sustained inhibitory effect. The incorporation of h‐BN may further regulate the release rate of Teb, thereby prolonging the inhibitory effect. For comparison, the antifungal activity of the Teb‐assembled DC supramolecule was also tested using the mycelium growth method, with the Teb content in the medium determined to be 0.095 mg L^−1^. The commercial Teb suspension (SC) was also used as a control and demonstrates lower antifungal activity rate. In sharp contrast, as shown in Figures S14e–i (Supporting Information), the inhibition rate of the free Teb is 59.13%, while the value of Teb@DC increase to be 71.52%, demonstrating that the DC supramolecular system also enhances Teb's antifungal activity.

**Figure 8:**
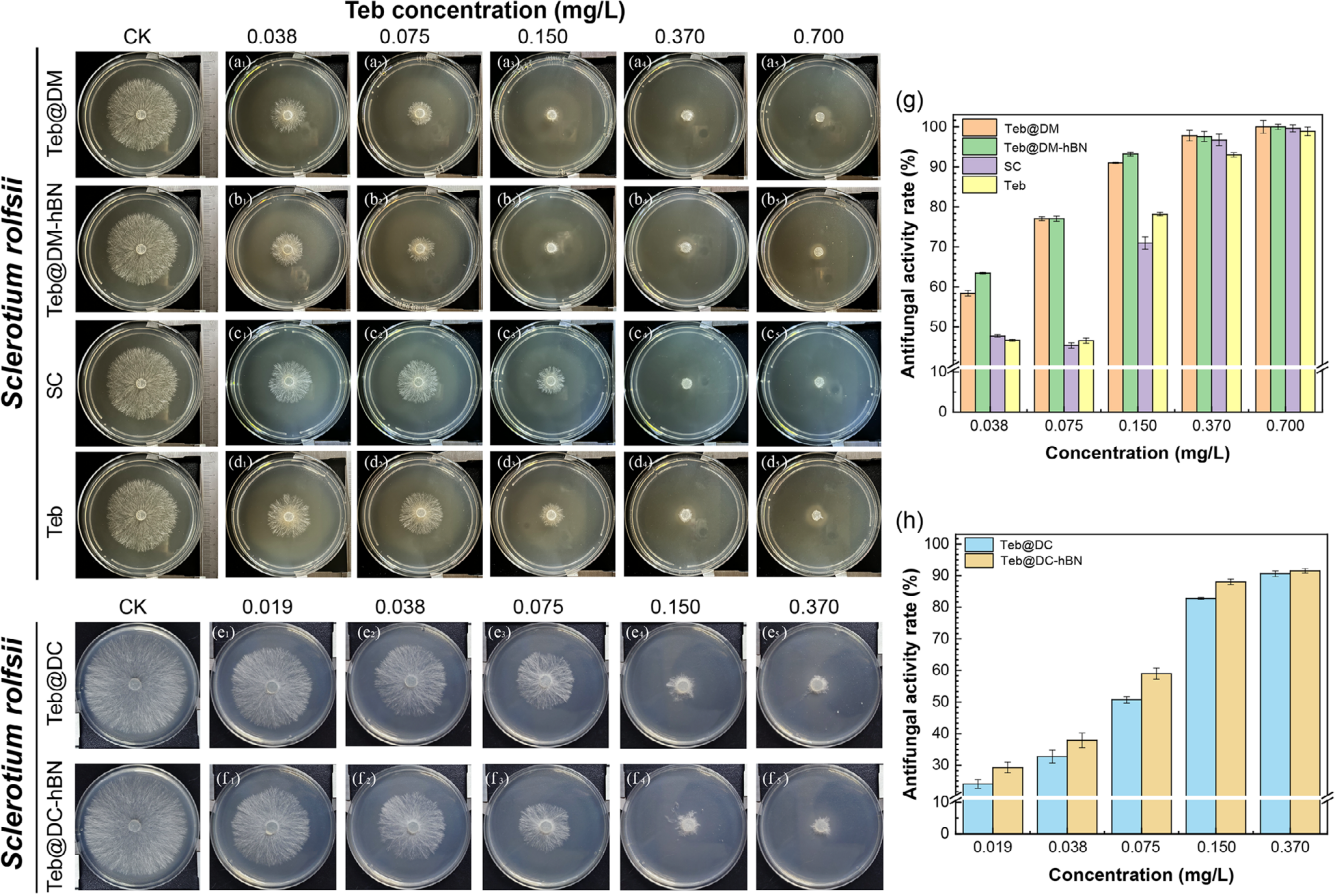
The digital images of the inhibition activity against *Sclerotium rolfsii* by Teb@DM a_1_–a_5_), Teb@DM‐hBN b_1_–b_5_), SC c_1_–c_5_), free Teb d_1_–d_5_), Teb@DC e_1_–e_5_), Teb@DC‐hBN f_1_–f_5_) at various Teb concentrations, g,h) the histogram of the antifungal activity rates of Teb@DM, Teb@DM‐hBN, SC, and Teb at various Teb concentrations.

Subsequently, the median effective concentration (*EC*_50_) values of Teb‐assembled supramolecules against *Sclerotium rolfsii* were determined as displayed in **Figure** **8** and Table S4 (Supporting Information). The *EC*_50_ values of Teb@DM and Teb@DC are calculated to be 0.033 and 0.053 mg L^−1^, respectively, obviously lower the values of SC and free Teb. In comparison, the antifungal activity of DM and DC supramolecular carriers were also measured as shown in Figure S15 (Supporting Information), increasing DM and DC concentrations significantly reduce hyphal growth and boost inhibition rates, indicating their inherent antifungal effects. This may be ascribed to hydrogen bonds between maleic acid or citric acids and DMPSA induced positively charged quaternary ammonium groups in DM and DC and then such cationic surfactant‐like structure disrupts fungal cell membranes. The small size and sustained‐release properties of Teb@DM and Teb@DC further improve their bioefficacy. Thus, DM and DC synergistically enhance Teb's antifungal activity. Notably, boron nitride may also synergistically enhance antifungal performance against soil‐borne fungal diseases, potentially by improving fungicide stability and release profiles. This allows lower Teb concentrations to achieve comparable antifungal effects, thus reducing the *EC*_50_ value.

### 2.13 Pot Experiment Validation

**Figure 9:**
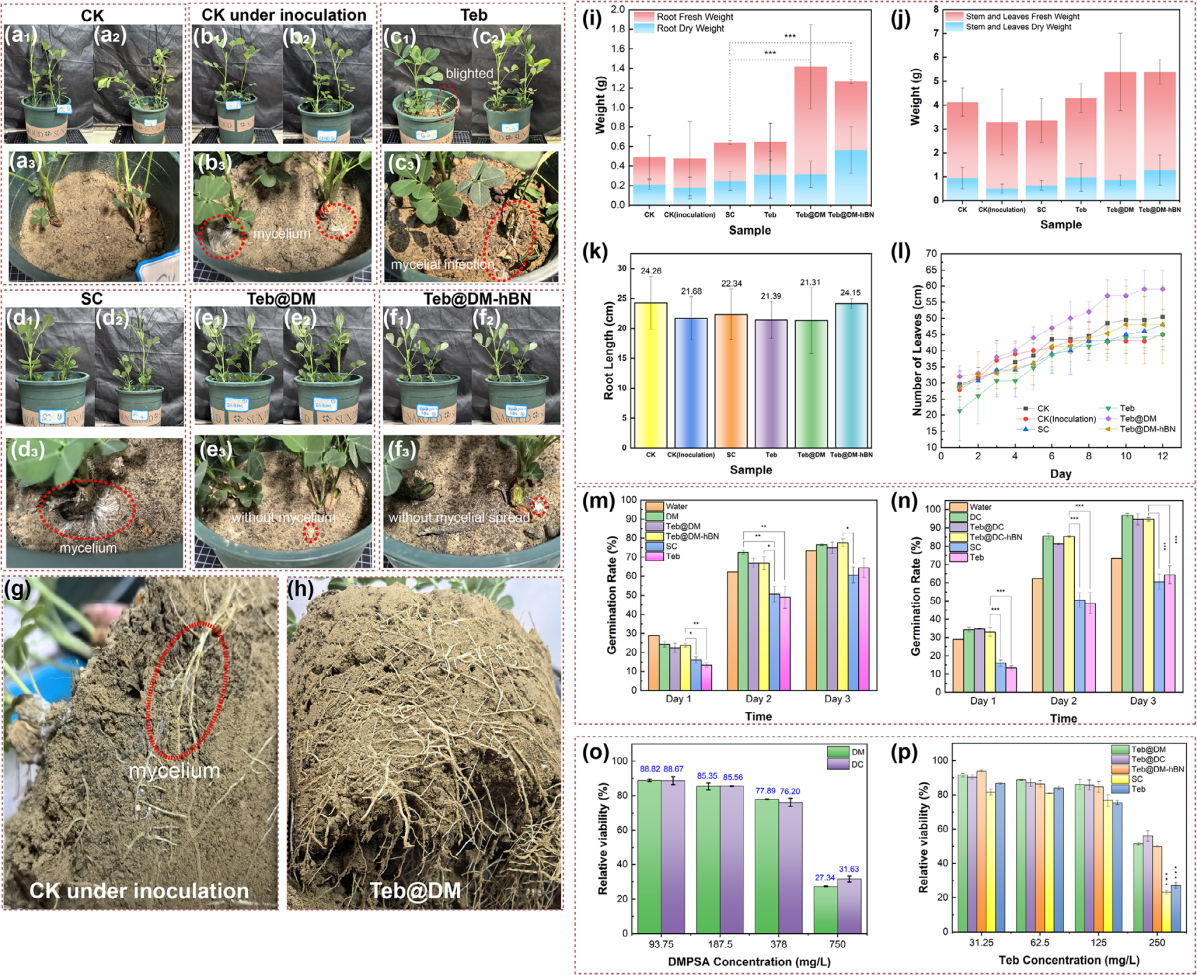
The growth conditions of peanut plants in pots at 21 days: a_1_–a_3_) CK without pathogenic inoculation, b_1_–b_3_) CK under pathogenic inoculation, c_1_–c_3_) Teb drenching, d_1_–d_3_) SC, e_1_–e_3_) Teb@DM, f_1_–f_3_) Teb@DM‐hBN against pathogen infection, including the fungal mycelia growth states on the surface of the potted plants, the peanut plant roots treated by g) CK and h) Teb@DM under pots soil with pathogenic inoculation for 21 days, i) the dry and fresh weights of peanut plant roots, j) the dry and fresh weights of peanut plant stems and leaves, k) the root length of peanut plant, l) the total number of leaf growth trend over the days. Germination rate of peanut seeds treated with m) DM, Teb@DM, Teb@DM‐hBN and n) DC, Teb@DC, Teb@DC‐hBN, using water, SC, Teb treatment as controls, over three days, o) the cytotoxicity of DM and DC on NIH3T3 cells at various DMPSA concentrations, p) the cytotoxicity of Teb‐assembled DM, DC, and DM‐hBN on NIH3T3 cells at a series concentrations of Teb using SC and Teb as controls (***: *p* ≤ 0.001, **: *p* ≤ 0.01, *: *p* ≤ 0.05).

To validate the efficacy of Teb‐assembled supramolecular systems in controlling peanut southern blight, taking Teb@DM and Teb@DM‐hBN as exemplars, the control efficacy against *Sclerotium rolfsii* in soil was continuously monitored for 21 days via root‐drenching method. As depicted in the **Figure** **9**b3, subsequent to pathogen inoculation, the mycelia of *Sclerotium rolfsii* in the blank group proliferate and expand within the soil over a 21‐day period, infiltrating the plant stems. Additionally, mycelial invasion is detected in the plant roots (Figure 9g). Similar phenomena are also evident in the free Teb treatment group (Figure 9c3) and the SC treatment group (Figure 9d3). This indicates that the inhibitory effects of Teb and SC against *Sclerotium rolfsii* are rather limited. Conversely, in the pot experiments subjected to root‐drenching treatment with Teb@DM and Teb@DM‐hBN, the spread of fungal mycelia is scarcely observable (Figure 9e_3_,f_3_). This can be ascribed to the synergistic antifungal enhancement of the supramolecular complex and active antifungal ingredient. Notably, following treatment with the Teb@DM supramolecular system, the peanut plant roots exhibit robust coiling growth and an increased number of branches (Figure 9h), which is conducive to peanut development. This may be associated with maleic acid, an endogenous plant substance that promotes plant growth.

Upon comparing the fresh and dry weights of the roots, it is found that both the fresh and dry weights of the roots in the pots treated with Teb@DM and Teb@DM‐hBN are significantly elevated (Figure 9i). The root lengths of the plants in the Teb@DM‐hBN treatment group are comparable to those of non‐pathogen inoculated blank plants, while the root lengths of the plants in the Teb@DM treatment group show a slight reduction (Figure 9k). In conjunction with the fresh and dry weight data of the roots, this further validates that the Teb@DM treatment stimulates more vigorous root growth and promotes root branching.

When observing the growth status of peanut plants, it was noted that one of the seedlings in the Teb group deteriorated (Figure 9c1), leading to a decline in the leaf count of the peanut plants (Figure 9l). The reduction in the number of leaves of the blank plants infected with *Sclerotium rolfsii* occurs on the 7th day, confirming the successful invasion of the pathogen in the non‐drenched peanut plants (Figure 9l). The peanut plants in the Teb@DM treatment group demonstrat the fastest leaf growth rate, indicating that they are not inhibited by the fungicide or affected by pathogen invasion. Moreover, both the dry and fresh weights of the stems and leaves of the plants treated with Teb@DM and Teb@DM‐hBN are higher than the case of the non‐pathogen inoculated blank group (Figure 9j).

In summary, Teb@DM and Teb@DM‐hBN can effectively combat the invasion of the fungal southern blight and promote the plant growth especially the plant root system, highlighting their potential as eco‐friendly fungicide carriers.

### 2.14 Biological Safety Evaluation

Teb usually exerts a certain phytotoxic impact on seed germination. As depicted in Figure 9m,n, the germination rates of seeds treated with the free Teb and the commercially Teb suspension concentrate (SC) are notably lower than those of seeds soaked in pure water.

By the third day, the germination rates merely reach ≈60%, suggesting that Teb significantly inhibits the growth of seeds during the germination stage. Conversely, the supramolecular carriers DM and DC demonstrat a promotional effect on seed germination, exhibiting higher germination rates than those of the pure water‐soaked seeds. Among them, the germination rate of DC was higher than the value of DM. On the third day, the germination rates of DM and DC exceed 75% and 95%, respectively. This phenomenon may be attributed to the water retention capacity of the supramolecular systems, which ensures water homeostasis during the seed germination period. Additionally, the porous network structure of the supramolecules enhances the respiratory metabolism of seeds. Moreover, the structural variations among supramolecules play a regulatory role in seed germination. After assembling the same concentration of Teb into the supramolecular carriers DM and DC, the germination rates are slightly lower than those of the supramolecular carriers alone, but are significantly higher than the case of seeds treated with the free Teb. This finding indicates that the supramolecular gels act as a shield, protecting seeds from the phytotoxic effects of Teb, thereby highlighting the excellent biocompatibility of the DM and DC supramolecular gels. Intriguingly, the treatments of Teb@DM‐hBN and Teb@DC‐hBN led to a slight increase in the seed germination rate compared to Teb@DM and Teb@DC without boron nitride. This can be attributed to the formation of a micro‐nano fence‐like structure around the seeds by the 2D sheets of h‐BN.

The cytotoxicity of the Teb‐assembled supramolecular systems was evaluated as presented in Figure 9o,p. The cytotoxicity of the DM and DC supramolecular carriers demonstrats a remarkable concentration‐dependent relationship. When the DMPSA concentration is within 378 mg L^−1^, the cell viability exceeds 75%, indicating relatively minimal cytotoxicity. However, as the concentration increases beyond this point, cell viability declines sharply, particularly within the 378–750 mg L^−1^ range, suggesting the presence of a critical toxicity threshold in this interval. The cytotoxicity profiles of DM and DC are attributed to the structural characteristics of DMPSA, exerting cytotoxic effects at high concentrations by compromising cell membrane integrity.

Likewise, the cytotoxicity of Teb also shows a distinct concentration‐dependence, which is in line with the previous findings regarding the impact of Teb on seed germination rates. At all tested concentrations, Teb@DM, Teb@DC, and Teb@DM‐hBN exhibits higher cell viability compared to Teb and SC. Specifically, at a concentration of 250 mg L^−1^, the cell viability of the cells treated with Teb@DM is approximately twice that of the cells treated with Teb. This indicates that the DM supramolecular carrier can effectively mitigate the cytotoxicity of Teb, via DM and DC supramolecular structures regulating the release of Teb and minimizing the contact of cells with high concentration Teb. Furthermore, under high concentration Teb conditions, the cell survival rate of Teb@DC is ≈4% higher than the case of Teb@DM, which is consistent with the trend observed in seed germination rates. Assembling Teb within the DM and DC supramolecular gels can significantly reduce its cytotoxicity. This approach not only maintains the efficacy of Teb but also substantially enhances its safety. Additionally, the incorporation of boron nitride inorganic materials does not introduce toxicity to the system, underscoring its biocompatibility and reinforcing the safety advantages of the system.

## 3 Conclusion

To address adhesion and persistence limitations of conventional fungicides, this study demonstrates that h‐BN reinforced supramolecular gels (Teb@DM‐hBN and Teb@DC‐hBN) represent a transformative strategy for sustainable management of soil‐borne fungal pathogens. By synergizing the microenvironmental responsiveness of low molecular weight gels with h‐BN's adhesive and structural properties, the nano‐assembled supramolecular gels overcome critical limitations of conventional fungicides. The incorporation of h‐BN into supramolecular gels synergistically enhances the mechanical stability. The leaf surface contact angle decreases by 52% compared to raw Teb, and the anti‐erosion performance is remarkably improved compared to other counterparts identifying with stronger fluorescence intensity. Soil interactions are tunable by h‐BN disrupted supramolecular shells, exposing Teb for moderate soil adsorption with K_d_ of 53.26 mL g^−1^, while extended soil retention time to 940 min, elevating14.69 times and 2.46 times compared with free Teb and non‐reinforced supramolecular gel. Teb@DM‐hBN achieves the retention capacity with 41.45% around peanut roots, higher than the case of Teb@DM (31.66%) and commercial formulation (13.48%). Teb@DM‐hBN boostes biological efficacy against *Sclerotium rolfsii* with *EC*_50_ decreased by 54.10%, and pot trials show complete mycelial suppression with promoted root branching. Cytocompatibility with ≈85% cell viability at 125 mg L^−1^ and seed germination rates with 75% matching controls confirm environmental safety. This work demonstrates of 2D material‐mediated stimuli‐responsive transport in soft matter systems, pioneers a new class of nano‐bridged carriers with mechanically strengthening and programmable permeability characteristics designed to overcome persistent barriers in soil‐borne disease management.

## 4 Experimental Section

### Materials

*N*‐[3‐(dimethylamino)propyl]stearamide (DMPSA, analytical grade) was purchased from Guangdong Wengjiang Chemical Reagent Co., Ltd. . Maleic acid (MA, ≥99%), citric acid (CA, ≥99.5%), hexagonal boron nitride (h‐BN, 99%, with the average size of 1‐2 µm), fluorescein isothiocyanate (FITC, 95%, a mixture of 5‐ and 6‐isomers), and stearyltrimethylammonium bromide (STAB, ≥98%) were sourced from Aladdin Co., Ltd. (Shanghai, China). The technical tebuconazole (Teb, 97.5%), pyraclostrobine (Pyr, 98.3%), thifluzamide (Thi, 96.0%), fluopyram (Flp, 99.6%), fluxapyroxas (Flx, 99.0%) were provided by Shenzhen Noposion Co., Ltd. . Sodium hydroxide (NaOH, 97%), hydrochloric acid (≥37%), and anhydrous ethanol (≥99.5%) were obtained from Damao Chemical Reagent Factory (Tianjin, China). Sodium hypochlorite solution (6–14% active chlorine) was purchased from Shanghai Macklin Biochemical Technology Co., Ltd. (Shanghai, China). Commercial tebuconazole suspension concentrate (SC, with concentration of 430 g L^−1^) was obtained from Shandong Bainongsida Bio Technology Co., Ltd. . Potato dextrose agar (PDA) was obtained from Beijing Solarbio Science & Technology Co., Ltd (Beijing, China). The hydroponic solution for plants was provided by Guangzhou Academy of Agricultural Sciences. Deionized water was used for all experiments, and all chemicals were used directly without further purification.

### Hexagonal Boron Nitride Reinforced Supramolecular Nano‐Assembled with Fungicide

Teb was chosen as the model fungicide. Based on the mass ratio of Teb to DMPSA being 1:3, with DMPSA accounting for 6% of the total mass, the molar ratio of DMPSA to MA or CA being 2:1, and boron nitride accounting for 1% of the total mass, a boron nitride reinforced supramolecular system assembled with Teb was prepared. In detail, a specific mass of DMPSA was weighed and placed into a conical flask. Then, a measured amount of deionized water was added to the flask, and the mixture was heated and stirred in a thermostatic magnetic stirrer at 70 °C. Subsequently, a precisely weighed quantity of MA or CA was measured according to the molar ratio. The acid was added to a certain amount of deionized water and thoroughly stirred to dissolve, forming an aqueous solution of MA or CA. A defined mass ratio of boron nitride powder and Teb was added to this solution. The mixture was then rapidly stirred at a rotational speed of 300 rpm using a homogenizer and subjected to ultrasonic treatment to form a uniformly dispersed fungicide‐containing dispersion of MA‐boron nitride or CA‐boron nitride. This dispersion was slowly and uniformly added to the DMPSA mixture. The assembly was maintained under continuous stirring and heating at 70 °C for 30 min. As a result, the boron nitride reinforced supramolecular gels assembled with Teb were obtained, which were designated as Teb@DM‐hBN and Teb@DC‐hBN, respectively. The gel‐state of Teb@DM‐hBN and Teb@DC‐hBN supramolecules is shown in the inset of Figure 1c,d, which can support its own weight during inverted. As a control, Teb‐assembled DM and DC supramolecules (marked as Teb@DM and Teb@DC) were prepared without adding h‐BN as described in Supporting Information.

### Characterizations

The sol‐state supramolecules were characterized using cryogenic scanning electron microscopy (Cryo‐SEM) for visualization and image acquisition (ZEISS Sigma 300, Germany). Transmission electron microscopy (TEM, JEOL JEM‐F200, Japan) was used for structure analysis of the boron nitride within supramolecular samples. The surface morphology, layers, and thickness of boron nitride collecting within supramolecular samples after washing and centrifugation were examined by atomic force microscopy (AFM) (Bruker Dimension Icon, Germany) in tapping mode. The particle size and zeta potential of the sol‐state supramolecules by diluted and dispersed in deionized water with adjusted the content of DMPSA of 0.06% were carried out using a laser particle size analyzer (90 Plus PALS, Bruker Corporation, USA) via dynamic light scattering (DLS) technique at 25 °C with a scattering angle of 90° and PALS zeta potential mode. Each measurement was repeated three times, and the average value was calculated. The conductivities of aqueous solutions of Teb, MA, CA, Teb‐MA, and Teb‐CA were measured using a conductivity meter (DDS‐11A, INASE Scientific Instrument Co., Ltd.) with the probe rinsed with deionized water and dried with filter paper before each measurement. The viscosity and rheological properties of the gel‐state supramolecules were measured using a rotational rheometer (Thermo HAAKE, Thermo Scientific) at a shear rate of 10 s^−1^ over a temperature range of 25–80 °C and also at 25 °C over a shear rate range of 10‐300 s^−1^. The prepared gel‐state supramolecules were subjected to freeze‐drying and subsequently utilized for structural characterization and microscopic morphological analysis. For the microscopic morphological observation, scanning electron microscopy (SEM) (Tescan Mira Lms, ZEISS) was employed after 45 s of sputtering with gold. Thermogravimetric analysis (TGA) was carried out on a TGA2 thermobalance (Mettler‐Toledo) under a nitrogen atmosphere with a flow rate of 20 mL min^−1^, heating from 40 to 600 °C at a rate of 10 °C min^−1^ to compare the thermal stability and intermolecular forces of supramolecules. Differential scanning calorimetry (DSC) was performed using a Q20 calorimeter (TA Instruments) under a nitrogen flow of 50 mL min^−1^, heating from −10 to 150 °C at 10 °C min^−1^ and then cooling back to −10 °C at the same rate for one cycle. Fourier‐transform infrared spectroscopy (FTIR) was performed using a Spectrum 100 instrument (PerkinElmer, USA) with KBr pellet technique to compare vibrational states between 4000 and 400 cm^−1^ at a resolution of 4 cm^−1^ for identifying chemical interactions. The ^1^H nuclear magnetic resonance (^1^H NMR) spectra of the supramolecules were recorded using a nuclear magnetic resonance spectrometer at 400 MHz (Bruker) to analyze the chemical structure of the supramolecules. X‐ray diffraction (XRD, Rigaku Smartlab, Japan) was used to characterize the crystalline phase changes of the samples with a Cu‐Kα radiation source (40 kV, 30 mA) at a scanning rate of 10°·min^−1^ in the 2θ range of 5°–90°. X‐ray photoelectron spectroscopy (XPS) was carried out with a K‐Alpha spectrometer (Thermo Scientific) under vacuum using monochromatic Al Kα X‐rays to obtain the information about chemical composition and bonding chemistry for the Teb‐DMPSA, Teb‐MA, and Teb‐CA solid mixtures and DM supramolecule.

### Determination of CMC Value

The critical micelle concentration (CMC) of the supramolecules was determined using a surface tension method via a contact angle goniometer (Theat, Switzerland Biolin), based on the procedure outlined in the literature with modifications.^[^ ^55^, ^56^ ^]^ The CMC values were measured by diluting the supramolecular samples to specific concentrations of DMPSA as listed in Table S1 (Supporting Information). The pendant drop method was employed to record the surface tension values of the samples within 90 s. The average of the surface tension values measured in the last 5 s was used as the experimental data. Each sample concentration was measured in triplicate.

### Soil Isothermal Adsorption Test

The sandy loam soil (2 g) passing through a 0.25‐mm sieve was accurately weighed and transferred into a conical flask with a stopper. Subsequently, A series of solutions containing various concentrations of Teb (50 mL) with 12.5, 25, 50, 100, 200, and 400 mg L^−1^, which were prepared using CaCl_2_ solution (0.01 mol L^−1^) to keep the ionic concentration, were added to ensure a constant water to soil mass ratio (25:1 w/w). The initial Teb content in Teb@DM, Teb@DM‐hBN, Teb@DC, and Teb@DC‐hBN was maintained consistent. The target concentrations were achieved through dilution for experimental use. The conical flask was then placed on a thermostatic shaker and shaken at a constant temperature of 25 °C for 24 h. After the shaking process, the soil suspension was transferred and centrifuged at 4000 rpm for 5 min. Then, the supernatant (5 mL) was pipetted into a new container, and methanol (5 mL) was added for extraction. The mixture was subjected to ultrasonic agitation for 5 min and then centrifuged at 6000 rpm for 5 min. After centrifugation, the supernatant was filtered through a 0.22‐µm organic filter and the Teb content was determined by HPLC to calculate the adsorption constant. All experimental treatments were performed in triplicate, and the average value was used as the experimental data. The soil adsorption data were processed following the steps described in Supporting Information.

### Soil Desorption Test

Soil adsorption experiments were conducted by centrifuging fungicide‐contained soil with a concentration of 200 mg L^−1^. Then, the entire supernatant was carefully decanted and separated, and the sediment at the bottom of fungicide‐contained soil was retained and then oven‐dried to a constant mass. The dried soil samples (200 mg) were precisely weighed and placed into a dialysis bag with a MWCO of 5000 Da. Subsequently, CaCl_2_ solution (5 mL, 0.01 mol L^−1^) was added to the dialysis bag. The dialysis bag was then securely sealed and transferred into a brown‐colored conical flask. Additionally, CaCl_2_ solution (45 mL, 0.01 mol L^−1^) was added to the conical flask, ensuring that the total volume of the solution within the flask reached 50 mL. After preparation, the conical flask was placed in a thermostatic shaker set at 30 °C. At specific time intervals, 1 mL of the solution outside the dialysis bag was carefully pipetted out. Immediately afterward, CaCl_2_ solution (1 mL, 0.01 mol L^−1^) was added to maintain the original volume. The aliquots of the sample solution (1 mL) were then subjected to analysis. By referring to the standard curve of the corresponding fungicide, the concentration of the fungicide in the sample solution was determined. Based on these measurements, the cumulative release percentage of the fungicide over time was calculated.

### Soil Retention Performance Test

The sandy loam soil passed through a 0.25‐mm sieve was filled into the column up to the 30 mL level. Cotton was placed at the bottom of the column to prevent soil exudation. Subsequently, Teb@DM, Teb@DM‐hBN, Teb@DC, and Teb@DC‐hBN supramolecular solutions (50 mL) were added separately, using pure Teb, and SC as control groups. Immediately after the addition of the sample solutions, a digital camera was used to record the dripping process until the last drop of liquid fell. The retention time was measured and documented.

### Antifungal Activity In Vitro Assay

The antifungal activity of the supramolecules against *Sclerotium rolfsii* was evaluated using the mycelial growth rate method. The isolated *Sclerotium rolfsii* was inoculated onto PDA plates and incubated at 28 °C for 60 h. Teb@DM, Teb@DC, Teb@DM‐hBN, and Teb@DC‐hBN were incorporated into the PDA medium, with pure Teb and SC as control groups. Based on preliminary experiments, a series concentrations of the Teb‐loaded PDA medium were set, with three replicates for each concentration. PDA medium without the addition of any fungicide served as the blank control (CK). The growth of the mycelium was monitored continuously over a period of 6 days. At a consistent position with the same diameter, a mycelial disc with a diameter of 7 mm was punched out. The mycelial‐side of the disc was placed face‐down at the center of the PDA plate. After sealing the edges of the petri dishes with parafilm, they were incubated in the dark at 28 °C for 60 h. The petri dishes were photographed, and the colony diameters of each treatment were measured using the cross‐intersection method with ImageJ software. The antifungal inhibition rate was calculated according to **Equation (1)**, where *L*_0_ represents the diameter of the fungal colony in the control group (CK), *L*_t_ represents the diameter of the fungal colony in the treated group, and *L*_1_ represents the diameter of the mycelial plug used for inoculation.

(1) $$\mathrm{Anti}\;\mathrm{fung}\;\mathrm{alac}\;\mathrm{tivi}\;\mathrm{tyra}\mathrm{te}\left(\%\right)=\frac{L_{0}-L_{t}}{L_{0}-L_{1}}\times 100$$

The fungicide‐assembled supramolecules to be assayed were proportionally blended with sterilized PDA medium to prepare a series of fungicide‐incorporated plates with a set of concentration gradients (a total of five gradients). The plates were inverted and incubated in a 28 °C for three days. The *EC*_50_ values were determined by fitting the inhibition rates to the corresponding fungicide concentrations using nonlinear regression analysis of the dose‐response curve.

## Conflict of Interest

The authors declare no conflict of interest.

## Supporting information



Supporting Information

## Data Availability

The data that support the findings of this study are available from the corresponding author upon reasonable request.
