# Supplementary material for: Boron Nitride Reinforced Supramolecular Gels Nano‐Assembled with Fungicides toward Soil‐Borne Fungal Disease Management
Source: Adv Sci (Weinh). 2025 Nov 19;13(7):e15855. doi: 10.1002/advs.202515855 (PMC12866808; doi:10.1002/advs.202515855)
Supplement: Supplementary file 1 — Supporting Information [file ADVS-13-e15855-s001.pdf]

Supporting Information

Boron Nitride Reinforced Supramolecular Gels Nano-Assembled with Fungicides Towards Soil-Borne Fungal Disease Management

Li Hao[#]*, *Jialin Zeng*[#], *Weixian Qiu*, *Jinhui Wang*, *Xiaoshan Huang*, *Jiachun Wu*, *Zhensong Weng*, *Ziting Yin*, *Hongjun Zhou**, *and Xinhua Zhou**

L. Hao, J. Zeng, W. Qiu, J. Wang, X. Huang, J. Wu, Z. Weng, Z. Yin, H. Zhou, X. Zhou

School of Chemical and Materials Engineering, Key Laboratory of Green Prevention and Control on Fruits and Vegetables in South China of Ministry of Agriculture and Rural Affairs, Innovative Institute for Plant Health, Zhongkai University of Agriculture and Engineering, Guangzhou, Guangdong, 510225, China

E-mail: haoli20@tju.edu.cn (L. Hao), hongjunzhou@163.com (H. Zhou), cexinhuazhou@163.com (X. Zhou)

[#]Li Hao and Jialin Zeng contributed equally to this work.

S1. Experimental Section

S1.1. Supramolecular Assembly Based on DMPSA and Polyprotic Acid

The supramolecular self-assembly reaction and the intermolecular interactions driven by hydrogen bonds were employed to construct a stable supramolecular network. The amide group of *N*-[3-(dimethylamino)propyl]stearamide (DMPSA, whose structural formula presented in Scheme S1) acts as the primary hydrogen-bond acceptor. It forms hydrogen bonds with the protons provided by the carboxylic acids of maleic acid (MA) or citric acid (CA). Building on the previous preparation methods developed by our research group,[1] during the supramolecular synthesis process, DMPSA constitutes 6% of the total mass, and the molar ratio of DMPSA to MA or CA is set at 2:1.

The detailed preparation procedure is as follows: Initially, a specific mass of DMPSA was placed in a conical flask, and a measured volume of deionized water was added. The mixture was then heated to 70 °C and stirred vigorously. As DMPSA transformed from a white solid into an oily liquid, it melted within the aqueous solution, resulting in a homogeneous mixture. Subsequently, a calculated mass of maleic acid or citric acid, based on the molar ratio, was dissolved in a certain quantity of deionized water. This solution was then slowly and uniformly added to the DMPSA mixture. The self-assembly reaction was carried out under continuous heating and stirring at 70 °C. In the initial phase, the incipient gel structure emerged after approximately 3 min of reaction. To ensure complete self-assembly, the heating and stirring were continued for an additional 30 min. Ultimately, a supramolecular gel featuring a stable three-dimensional network structure was successfully obtained. During this process, through hydrogen-bond interactions, DMPSA and MA form a supramolecular entity designated as DM, while DMPSA and CA interact to generate a supramolecular species named DC. The gel-state of DM and DC supramolecules is shown in the inset of Figures S1b&c.

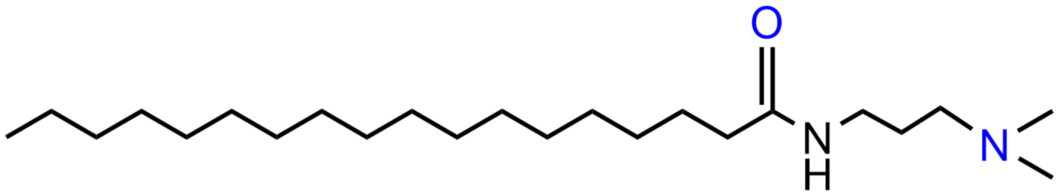

Scheme S1. The chemical structure of DMPSA.

S1.2. Hexagonal Boron Nitride Reinforced Supramolecular Assembly Based on DMPSA and Polyprotic Acid

The construction of a stable supramolecular network was achieved through hydrogen bond mediated interactions, leveraging supramolecular self-assembly reactions and intermolecular forces driven by hydrogen bonds. The amide group of DMPSA (whose structural formula is depicted in Scheme S1) acts as the primary hydrogen-bond acceptor. It forms hydrogen bonds with the protons furnished by the carboxylic acids of maleic acid (MA) or citric acid (CA). In the formulation, DMPSA accounts for 6% of the total mass, with a molar ratio of DMPSA to MA or CA setting 2:1 and the content of h-BN determining to be 1%.

The detailed preparation procedure is as follows: Initially, a specific quantity of DMPSA powder was combined with a measured amount of deionized water. The mixture was then subjected to thorough agitation at 70 °C, enabling DMPSA to melt within the aqueous medium and form a homogeneous mixture. Subsequently, a precisely weighed amount of MA or CA, determined according to the molar ratio, was dissolved in a certain volume of deionized water. A specific mass of boron nitride powder was added to this solution. The resulting mixture was rapidly stirred at a rotational speed of 300 rpm using a homogenizer for a specific duration and then treated with ultrasonic irradiation to form a uniformly dispersed MA-boron nitride or CA-boron nitride dispersion. This dispersion was then slowly and uniformly introduced into the DMPSA mixture. The reaction was maintained under continuous stirring and heating at 70 °C. During the initial phase of the reaction, a gel-like precursor emerged within approximately 3 min. To ensure complete self-assembly, the heating and stirring were continued for an additional 30 min. Eventually, supramolecular gels supported by

boron nitride, featuring a stable three-dimensional network structure, were successfully synthesized and designated as DM-hBN and DC-hBN, respectively. As a control, the supramolecules were also prepared without h-BN supported to form two types of supramolecular hydrogels (DM and DC) as stated in Supporting Information.

S1.3. Supramolecular Assembled with Various Fungicide

Teb was chosen as the model fungicide. Based on the mass ratio of Teb to DMPSA being 1:3, with DMPSA accounting for 6% of the total mass, the molar ratio of DMPSA to MA or CA being 2:1, Teb-assembled supramolecular system was prepared, marked as Teb@DM and Teb@DC. The gel-state of Teb@DM and Teb@DC supramolecules is shown in the inset of Figures 1a&b. Additionally, other fungicides including pyraclostrobine (Pyr,), thifluzamide (Thi), fluopyram (Flp), and fluxapyroxas (Flx) (whose structural formula displayed in Scheme S2) were also loaded using DM supramolecules as comparisons, designated as Pyr@DM, Thi@DM, Flp@DM, and Flx@DM.

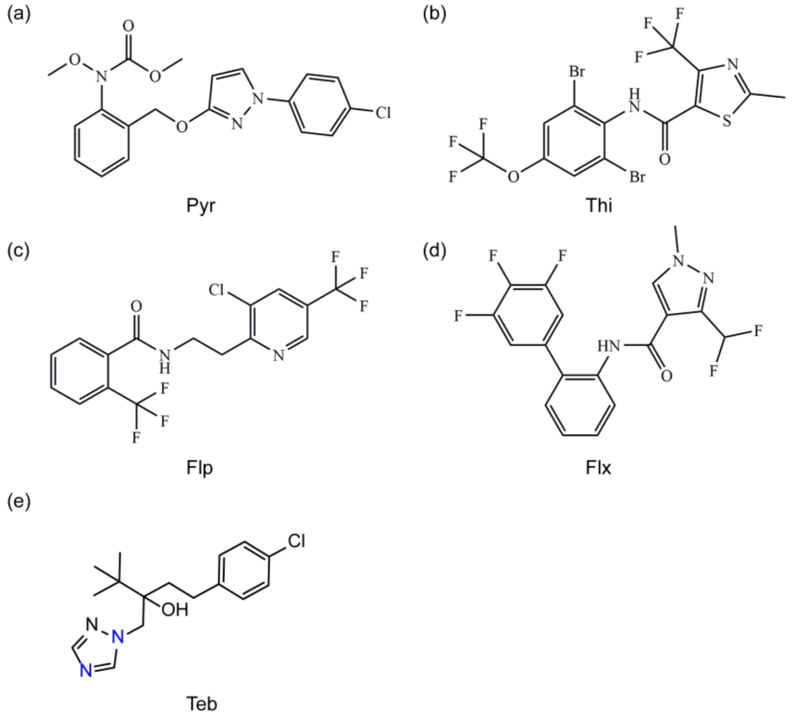

Scheme S2. The chemical structure of (a) Pyr, (b) Thi, (c) Flp, (d) Flx, (e) Teb.

S1.4. Characterizations

The sol-state supramolecules were characterized using cryogenic scanning

electron microscopy (Cryo-SEM) (Sigma 300, ZEISS). The synthesized supramolecules were dispersed in deionized water to form a homogeneous suspension. A suitable volume of this suspension was carefully pipetted onto a copper grid, ensuring uniform coverage of the grid's surface. Immediately afterwards, the grid was rapidly plunged into liquid nitrogen for cryo-fixation. This step was crucial for preserving the native structure of the supramolecules and preventing any structural alterations during subsequent handling. The cryo-fixed supramolecular samples were first subjected to preliminary drying in a freeze-dryer to eliminate excess surface moisture. Subsequently, the samples were fractured under liquid nitrogen conditions. This fracturing process was carried out to expose the internal architecture of the supramolecules. To enhance the electrical conductivity of the samples for optimal imaging in the electron microscope, a thin layer of gold was sputtered onto the samples. Finally, the prepared samples were loaded into the Cryo-SEM for visualization and image acquisition.

The viscosity and rheological properties of the gel-state supramolecules were measured using a rotational rheometer (Thermo HAAKE, Thermo Scientific). For the measurement of viscosity as a function of shear rate, the parameters were set with a shear rate ranging from 150-300 s^{-1}, while maintaining the temperature at 25 °C. Regarding the pH-responsive viscosity testing, 10 mL of HCl aqueous solution with a pH of 3, deionized water with a pH of 7, or NaOH aqueous solution with a pH of 11 were separately added to 50 mL of the supramolecular hydroges. After vigorous stirring for 10 min, the samples were allowed to stand for 2 h to obtain the supramolecular hydrogels with adjusted pH values. For the temperature-responsive viscosity measurement, the shear rate was fixed at 10 s^{-1}, and the temperature was varied within the range of 25-80 °C.

The conductivities of aqueous solutions of Teb, MA, CA, Teb-MA, and Teb-CA were measured using a conductivity meter (DDS-11A, INASE Scientific Instrument Co., Ltd.) with the probe rinsed with deionized water and dried with filter paper before each measurement. In the mixed solutions of Teb-MA or Teb-CA, the concentrations were maintained at levels consistent with the respective contents of Teb, MA, or CA. All samples were measured in triplicate (in parallel), and the final reported values were

the average of these measurements.

The prepared gel-state supramolecules were subjected to freeze-drying and subsequently utilized f or structural characterization and microscopic morphological analysis.

For the microscopic morphological observation, scanning electron microscopy (SEM) was employed (Tescan Mira Lms, ZEISS). The freeze-dried supramolecules were evenly distributed on the conductive adhesive. The samples were allowed to dry naturally at room temperature and then underwent gold sputtering for 45 s. The microscopic morphology of the freeze-dried supramolecules was examined under an accelerating voltage of 5 kV.

S1.5. Loading Capacity of Fungicides within Supramolecules

In order to determine the loading capacity of fungicides within supramolecules, the fungicide-loaded supramolecules gels were converted into a solution. The solutions were then centrifuged at 10000 rpm for 10 min. Subsequently, 1 mL of the supernatant was carefully pipetted and transferred into a brown volumetric flask. The volume was then adjusted to 10 mL using methanol solution. The collected supernatant was filtered through a 0.25-μm filter membrane and transferred into a vial suitable for liquid chromatography analysis. The fungicide content was quantified using high-performance liquid chromatography (HPLC, Agilent 1200). The chromatographic conditions were as follows: a Shim-pack GIST C18 reversed-phase column was used, the mobile phase consisted of a mixture of methanol and water at a ratio of 80:20 (v/v), the column temperature was maintained at 40 °C, the injection volume was 10 μL, and the pump flow rate was set at 1 mL min^{-1}. Based on the following HPLC standard curves equations of the five fungicides relating the concentration (C, mg L^{-1}) and peak area (A) (Teb: A=9533C+22832, Pyr: A=9303C+122145, Thi: A=23172C-151499, Flp: A=43356C-488907, Flx: A=21648C+194932), the encapsulation efficiency (*EE*, %) and the loading capacity (*LC*, %) of the five fungicides within the supramolecular carriers were calculated using Equation (S1&S2), where m_{carrier} (mg) indicates the mass of fungicide-loaded supramolecules, $m_{\mathrm{total\ AI}}$ (mg) represents the total quality of fungicides, $m_{\mathrm{free\ AI}}$ (mg) stands for the weight of free fungicides.

$$EE(\%) = \frac{M_{total\,AI} - m_{free\,AI}}{M_{total\,AI}} \times 100 \quad \text{(S1)}$$

$$LC(\%) = \frac{M_{AI} - m_{AI}}{M_{carrier}} \times 100 \quad \text{(S2)}$$

S1.6. Stimuli-Responsive Sustained Release Behavior

The release behavior of Teb from supramolecules was investigated under various pH and temperature conditions. Specific amounts of fungicide-loaded supramolecules were placed in dialysis bags (MWCO = 5000 Da). The bags were filled with 5 mL of 40% (v/v) ethanol-water solution and immersed in 45 mL of the same solution in brown conical flasks. The flasks were placed on a shaker and oscillated at 200 rpm. At predetermined intervals, 1 mL from the reservoir was collected and placed into a volumetric flask, and then diluted to 10 mL with a 40% ethanol-water solution. An equal volume of the corresponding medium was added back into the conical flask. Sustained release experiments were conducted at various temperatures (25, 40, and 55 °C, with pH=7) and pH values (3, 5, 7, 9, and 11, at 25 °C) to study the temperature- and pH-responsive release behavior of the fungicide-loaded supramolecular gels. The released amount of Teb was measured by HPLC using a calibration curve (A=-1168C+625). The cumulative release percentage (R_i) was calculated using Equation (S3), where, c_i represents the concentration of Teb in the release medium at a specific time point (mg L^{-1}), and m_{Teb} represents the total mass of the fungicide-loaded into the dialysis bag (mg).

$$R_i = \begin{cases} c_i \times 0.05/m_{Teb}(i = 1) \\ c_i \times 0.05/m_{Teb} + \sum_{i=1}^{i-1} c_i \times 0.001/m_{Teb}(i > 1) \end{cases} \quad \text{(S3)}$$

S1.7. Determination of CMC Value

In accordance with Table S1, an appropriate amount of water was added to each sample to dilute it to the corresponding DMPSA concentration.

Table S1. The dilution method of the supramolecules in determining surface tension.

No.	Concentration (mmol L^{-1})	Total volume of solution (mL)	The amount of added water (mL)
1	0.634	10.00	0.00
2	0.587	10.81	0.81
3	0.539	11.76	0.95
4	0.492	12.90	1.14
5	0.444	14.28	1.38
6	0.397	15.99	1.71
7	0.349	18.17	2.18
8	0.302	21.03	2.86
9	0.254	24.96	3.93
10	0.207	30.70	5.74
11	0.159	39.87	9.17
12	0.112	56.86	16.99
13	0.064	99.06	42.20
14	0.043	148.83	49.76
15	0.032	198.13	49.30

S1.8. Wetting Ability on Foliar Surfaces

Using the hydrophobic cucumber leaf surface as a model, the dynamic contact angles of sol-state DM, Teb@DM, Teb@DM-hBN, DC, Teb@DC, and Teb@DC-hBN on the hydrophobic leaf surfaces were investigated to evaluate the wetting properties of the supramolecular systems. Forty-day-old cucumber leaves cultivated in the laboratory were selected. The regions with fewer leaf veins were cut out and fixed onto glass slides. Based on the measured CMC values, the dilution concentrations of the samples were chosen to be close to the CMC values for wetting property measurements. The samples were diluted to 0.159 mmol L^{-1}, 0.079 mmol L^{-1}, and 0.036 mmol L^{-1} according to the DMPSA content. A micro-pipettor was used to drop 5.5-6 μL of the aqueous solutions onto the cucumber leaves. Immediately after contact, the dynamic contact angles of the samples on the leaf surfaces within 90 s were recorded using a contact angle goniometer (Theat, Switzerland Biolin). Water and SC were set as control groups, and the stable contact angle data were recorded. The contact angle values reported for each sample were the average of three measurements on three different batches of samples.

S1.9. Adhesion Characteristics on Foliar Surfaces

The adhesion performance of supramolecular solutions on hydrophobic leaves was

assessed by diluting the supramolecular gels to 0.5 mmol L^{-1} based on the DMPSA content. Similarly, the control solution (STAB) was prepared at 0.5 mmol L^{-1}. Cucumber leaves were cut into squares of the same area. They were dipped into the supramolecular solution for 10 s using tweezers, then vertically lifted out. Once the droplets stopped falling from the leaves, the weight of the leaf surface adhered by the supramolecules was measured. Each sample group was tested in ten parallels, and the liquid holding capacity (LHC) per unit area was calculated using the equation below, where M_0 and M_1 represent the mass of the cucumber leaf before and after immersion (mg), respectively, and A represents the area of the leaf (cm^2).

$$LHC = \frac{M_1 - M_0}{A} \quad \text{(S4)}$$

S1.10. Impact Experiment

Before the experiment, the microliter syringe was mounted on a bracket. The polytetrafluoroethylene (PTFE) membrane surface was pasted onto the slide. The entire process of the droplet impacting the PTFE surface was meticulously recorded using a high-speed camera (Phantom, VEO-410L) at the rate of 5200 frames per second.

S1.11. Assessment of Anti-Erosion Performance

Fluorescein-labeled fungicide-assembled supramolecular samples were obtained by incorporated 20 mg of fluorescein isothiocyanate (FITC). The concentration of FITC in the fungicide-assembled supramolecular systems was set at a fixed value of 0.4 mg mL^{-1}, and the content of Teb in each sample was maintained constant. The control groups included pure Teb and a commercial Teb suspension concentrate (SC). The fluorescently labeled fungicide-assembled supramolecular system was diluted 200-fold to prepare a supramolecular dilution solution containing FITC for spraying applications. One milliliter of this fluorescently labeled supramolecular dilution was evenly sprayed onto cucumber leaves and allowed to dry naturally. Subsequently, 5 mL of deionized water was sprayed onto the leaves at an angle of 60° for 30 s to simulate rainwater erosion. After the water droplets had dripped off and the leaves had dried, the fluorescence intensity in the leaf surfaces and leaf vein regions before and after the scouring process was observed using an inverted fluorescence microscope (Dmi8,

Leica). This approach was employed to assess the anti-erosion performance of the fungicide-loaded supramolecular systems.

S1.12. Soil Isothermal Adsorption Test

Adsorption percentage (*A*, %) and the amount of the supramolecules adsorbed in the soil (*x*, μg) were calculated via Equations (S5&6), where M is the mass of the test substance in the aqueous solution without soil (μg), C_e is the equilibrium concentration of the test substance in the aqueous phase (μg mL^{-1}), V_0 is the volume of the water phase (mL).

$$A = \frac{100(\mathrm{M} - \mathrm{C_e} \times \mathrm{V_0})}{\mathrm{M}} \quad \text{(S5)}$$

$$x = \frac{M \times A}{100} \quad \text{(S6)}$$

Soil adsorption coefficient and the adsorption behavior of sandy loam soil for the test substance was determined using the Freundlich equation (S7&8), where C_s is the adsorption content of the test substance in sandy loam soil (μg g^{-1}), K_f is the Freundlich soil adsorption coefficient, m is the mass of sandy loam soil (g), and 1/n is the slope of the C_s versus C_e relationship curve. And the Equation (S7) can be transformed into the format of Equation (S9).

$$\mathrm{C_S} = \mathrm{K_f} \times \mathrm{C_e^{\frac{1}{n}}} \quad \text{(S7)}$$

$$\mathrm{C_S} = x/\mathrm{m} \quad \text{(S8)}$$

$$\ln C_s = \ln K_f + \frac{1}{n} \ln C_e \quad \text{(S9)}$$

Besides, the soil adsorption behavior of the sample was also described using a linear adsorption isotherm as Equation (S10), where K_d is the soil adsorption coefficient (mL g^{-1}).

$$\mathrm{C_S} = \mathrm{K_d} \times \mathrm{C_e} \quad \text{(S10)}$$

The adsorption of the test substance in soil is significantly influenced by soil organic matter. The soil adsorption coefficient normalized to organic carbon (K_{oc}) was calculated using Equation (S11), where K_{oc} is the soil adsorption coefficient based on organic carbon content (mL g^{-1}), and OC is the organic carbon content of the soil (%). For sandy loam soil, OC was assumed to be 1%.

$$K_{OC} = \frac{100K_d}{OC} \quad (S11)$$

The change in soil adsorption free energy (ΔG) was calculated using Equation (S12), where ΔG is the change in adsorption free energy ($kJ\cdot mol^{-1}$), R is the molar gas constant ($8.314\ J\ K^{-1}\ mol^{-1}$), and T is the absolute temperature (K).

$$\Delta G = -RT\ lnK_{oc} \quad (S12)$$

S1.13. Root Adhesion Capability Measurement

In accordance with the method reported in the literature,[2] on the fifth day following peanut germination, when the peanut plants had developed roots approximately 2-3 cm in length, each germinated peanut was individually subjected to hydroponic cultivation in a small bottle containing a plant growth nutrient solution (as depicted in Figure S1). After 16 days of growth, these plants were utilized for testing the root adhesion performance. The peanut roots were first thoroughly rinsed. Subsequently, they were immersed in various supramolecular solutions, with a concentration set at $0.6\ mg\cdot mL^{-1}$ for a duration of 10 s. As control groups, the roots were immersed in either pure water or a dispersion of the Teb active ingredient (SC). The root adhesion performance was quantified using the mass weighing method. Prior to weighing, the cleaning solution and any residues on the root surfaces were carefully blotted dry with absorbent paper. After the immersion period, the roots were suspended for 10 s. Once the liquid droplets on the roots had ceased to drip, the roots with adhered samples were weighed. Each sample group was subjected to ten parallel measurements. The liquid retention capacity (LRC, %) was calculated in accordance with Equation (S13), where M_0 and M_1 represent the mass of the root before and after soaking (mg).

$$LRC(\%) = \frac{M_1 - M_0}{M_0} \times 100 \quad (S13)$$

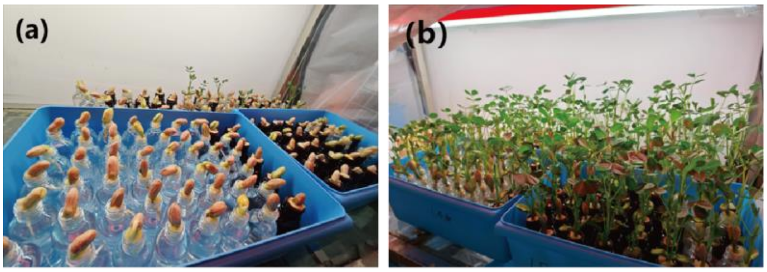

Figure S1. Digital photographs of peanut hydroponic cultivation stages.

S1.14. Seed Germination Evaluation

In accordance with the method reported in the literature,[3] peanut seeds were disinfected by immersion in a 1% sodium hypochlorite solution for 20 min. Subsequently, the surfaces of the peanut seeds were repeatedly rinsed with deionized water. The seeds were then air-dried naturally at room temperature. The treated seeds were immersed for 12 h respectively into Teb@DM, Teb@DM-hBN, Teb@DC, Teb@DC-hBN aqueous dispersions using SC and Teb as controls, all of which contained the same amount of Teb. The DM and DC supramolecules were established following the dilution protocol of Teb@DM and Teb@DC. Each group consisted of 25 seeds, and the experiment was replicated three times in parallel. Deionized water was employed as the experimental control. After immersion, the surface moisture of the peanut seeds was air-dried at room temperature, and the seeds were then placed in sterile germination boxes. Every day, deionized water (3 mL) was sprayed onto the seeds, and the boxes were incubated in a constant temperature incubator maintained at 27 °C. The number of germinated seeds was counted daily starting from the first day. The germination rate (GR) was calculated using Equation (S14), where N_0 denotes the total number of peanut seeds in the initial experiment, and N_i represents the number of germinated seeds treated by the sample group.

$$\mathrm{GR}(\%) = \frac{N_i}{N_0} \times 100\% \quad \text{(S14)}$$

S1.15. Cytotoxicity Assay

The cytotoxicity of five samples on NIH3T3 cells was evaluated using the MTT assay. NIH3T3 cells in the logarithmic growth phase were trypsinized, and the cell suspension was seeded into 96-well plates at a density of 5×10^3 cells per 100 μL, incubated at 37°C with 5% CO_2 for 8 h. After incubation, the medium was replaced with various sample concentrations. For Teb-containing samples, six concentrations (250, 125, 62.5, 31.25 mg L^{-1}) were tested, while for DM and DC supramolecules, six concentrations based on DMPSA content (750, 375, 187.5, 93.75 mg L^{-1}) were used. Each concentration was tested in triplicate with a blank control group containing only fresh medium. After 24 h, the medium was removed, cells were washed twice with PBS,

and 100 μL of MTT solution was added per well, followed by incubation at 37 °C for 4 h. The MTT solution was then replaced with 150 μL DMSO, and the plate was shaken at 37 °C and 400 rpm for 10 min. Absorbance was measured at 570 nm using a microplate reader. Cell viability rates were calculated relative to the control group set at 100% viability, using Equation (S15), where OD_i represents the optical density of cells treated with the sample, and OD_{ck} represents the optical density of untreated control cells.

$$\mathrm{CR}(\%) = \frac{OD_i}{OD_{ck}} \times 100\% \quad \text{(S15)}$$

S1.16. Pot Experiment for Plant Growth

To further investigate the control efficacy of the fungicide-loaded supramolecular system against *Sclerotium rolfsii* within peanut plants, a pot experiment was carried out. Initially, peanut seeds were soaked in 1% sodium hypochlorite solution for 20 min, then rinsed and soaked in deionized water for 12 h to induce germination. After the germination period, 8-day-old seedlings were transplanted into pots, with four replicates per group. For the first 2 days after transplantation, only watering was performed. Once the seedlings stabilized, oat grains inoculated with *Sclerotium rolfsii* mycelium were placed around the peanut stems. Various sample solutions with a Teb content of 78 $mg \cdot L^{-1}$ were prepared. Then, each sample solution (5 mL) was sprayed onto the peanut low stems near the roots. Subsequent applications were done every five days via root drenching. The plants were cultivated in a constant-temperature incubator under the following conditions of a 12:12 light-dark cycle, a temperature of 28 °C, and a humidity of 80%. Peanut seedlings without any treatment and peanut seedlings inoculated with *Sclerotium rolfsii* served as the control groups. The growth status and height changes of the plants were observed at regular intervals. The growth conditions were continuously monitored and recorded 21 days after seed sowing. The fresh and dry weights of the plant roots, stems, and leaves were measured, and the root length of the plants was also determined.

S1.17. Statistical Analysis

The data was analyzed by the one-way ANOVA followed by Tukey post-hoc test's

multiple comparison tests ($p < 0.05$) and expressed as the mean ± standard deviation (SD) for all the experiments using Origin software.

S2. Results and Discussion

S2.1. Morphological and Structural Characterization

DMPSA contains long hydrophobic carbon chains, which tend to aggregate to minimize contact with water or other polar molecules, meanwhile, such hydrophobic interaction facilitates the spontaneous arrangement of molecules into two-dimensional lamellae (Figure S2a).

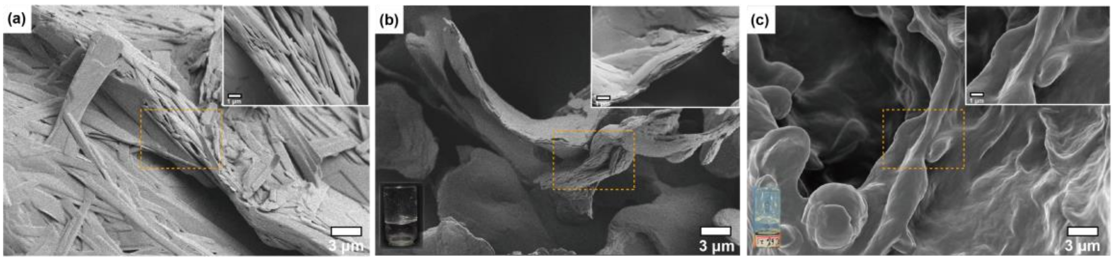

Figure S2. SEM images of (a) DMPSA, and freeze-dried supramolecular gel of (b) DM and (c) DC, where left corner insets displaying digital photos of corresponding inverted gel state supramolecules and right corner insets showing corresponding SEM images with higher magnifications.

Figure S3 depicts photographic images demonstrating the gel-sol transition state of the Teb@DM-hBN supramolecular system. At pH ~6.5, the system exists as a stable gel that does not flow upon vial inversion. Upon acidification, it undergoes a clear phase transition to a free-flowing sol state. Furthermore, this gel-sol transition is reversible over multiple cycles by alternately adjusting the pH.

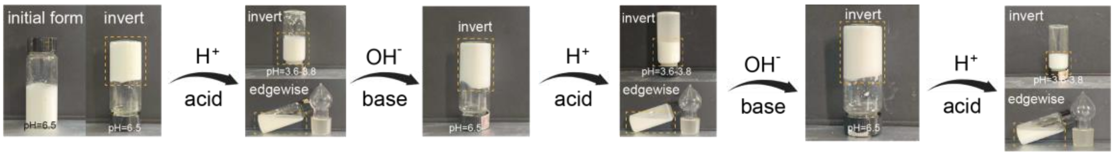

Figure S3. pH responsive gel-sol transition by adding acid or base to specific pH values.

Atomic force microscope (AFM) measurement confirms the dimensions of the h-BN nanosheets dispersed within the gel matrix in Figure S4. The results indicate a sheet thickness of approximately 15-20 nm and lateral sizes ranging from 80 to 150 nm.

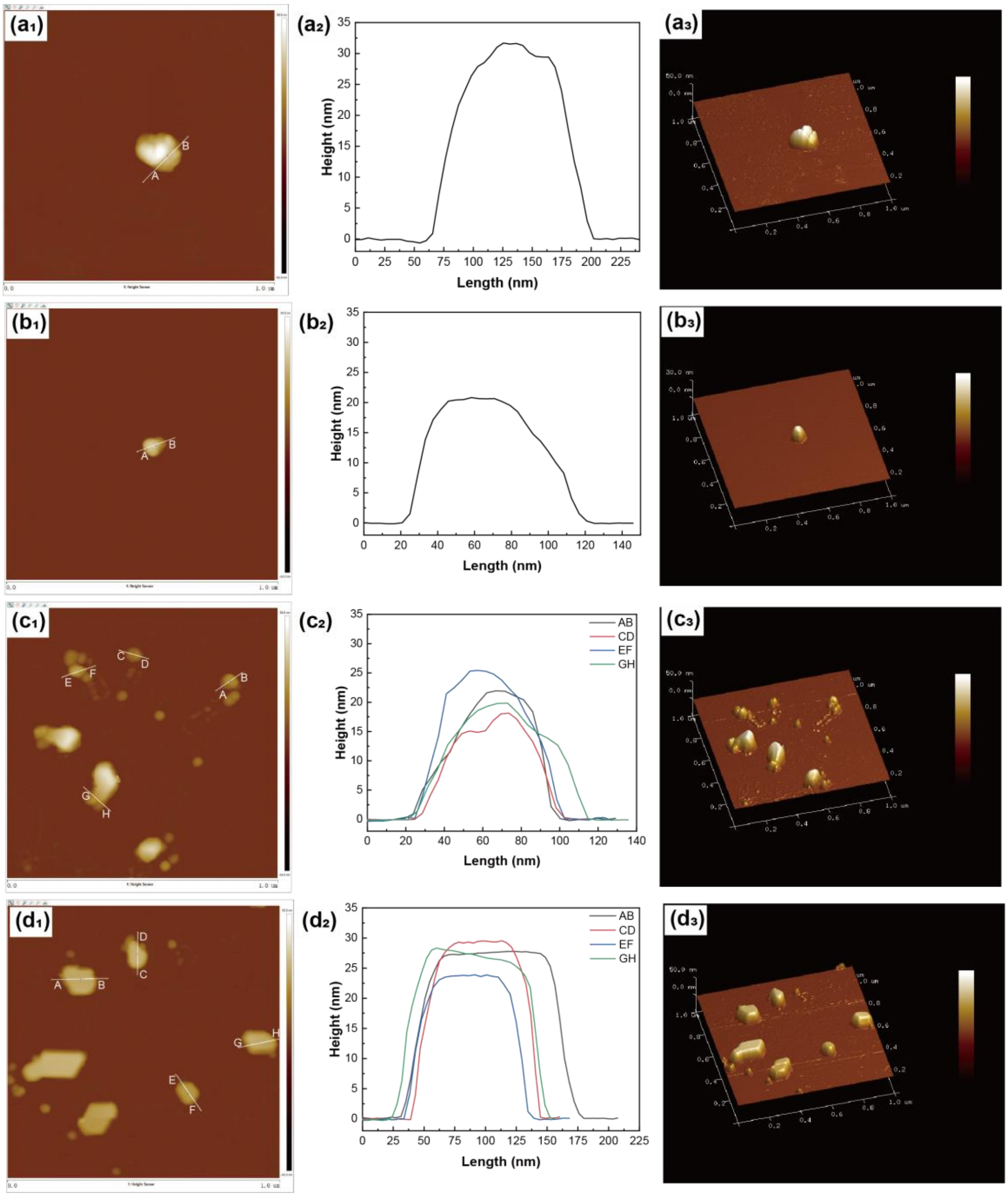

Figure S4. AFM 2D topography maps, height profiles, and 3D topographical images of boron nitride nanosheets collecting within (a_1-a_3) DM-hBN, (b_1-b_3) Teb@DM-hBN, (c_1-c_3) DC-hBN, and (d_1-d_3) Teb@DC-hBN after washing and centrifugation.

Transmission electron microscope (TEM) and corresponding HR-TEM imaging in Figure S5 reveal lamellar structures and clear lattice fringes, providing direct evidence of the well-defined crystalline structure of the incorporated h-BN.

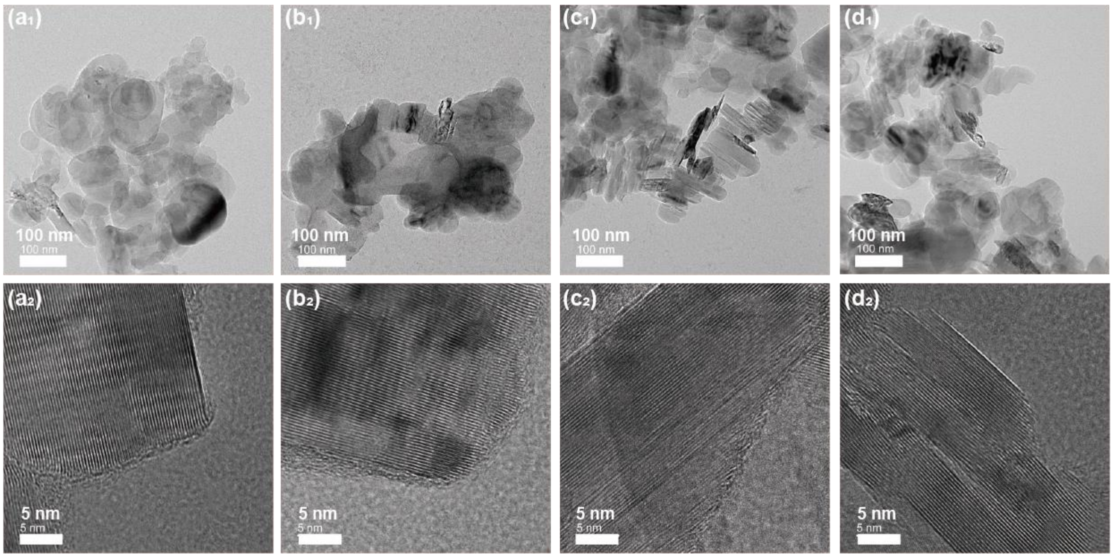

Figure S5. TEM and HR-TEM images of boron nitride nanosheets collecting within (a_1-a_2) DM-hBN, (b_1-b_2) Teb@DM-hBN, (c_1-c_2) DC-hBN, and (d_1-d_2) Teb@DC-hBN after washing and centrifugation.

S2.2. Thermodynamical Behavior

As revealed by comparative thermogravimetric analysis (Figures S6a&b), both DM and DC exhibit reduced thermal decomposition peaks compared to pure DMPSA (314 °C), falling to 311 °C and 298 °C, respectively. This decrease is attributed to the supramolecular structure weakening C-C bond cleavage in the DMPSA carbon backbone, indicating that DC requires less energy for chain scission than the case of DM. Additionally, new thermal decomposition peaks emerge within DM and DC, located at 218 °C and 187 °C, respectively. The lower decomposition temperature of DC (187 °C vs. 218 °C for DM) implies that minimal heat input can trigger partial disintegration. Furthermore, various fungicide-assembled DM gels (Pyr@DM, Thi@DM, Flp@DM, Flx@DM) also show the reduced decomposition peaks compared to DM (Figures S3d-g).

Compared with DMPSA and DM in Figure S6c, during the heating process, DMPSA exhibits only single endothermic peak (70.72 °C) attributed to main chain phase transition, while DM splits into two endothermic peaks at 56.56 °C and 45.79 °C indicative of two distinct hydrogen bond types between MA and DMPSA. During the cooling process, DMPSA possesses only one exothermic peak, which appears large hysteresis at 61.75 °C, and DM also has only one exothermic peak, which drops to

52.19 °C with reduced hysteresis. Moreover, other fungicide-assembled DM gels also exhibit thermally recyclable property (Figure S6h-k).

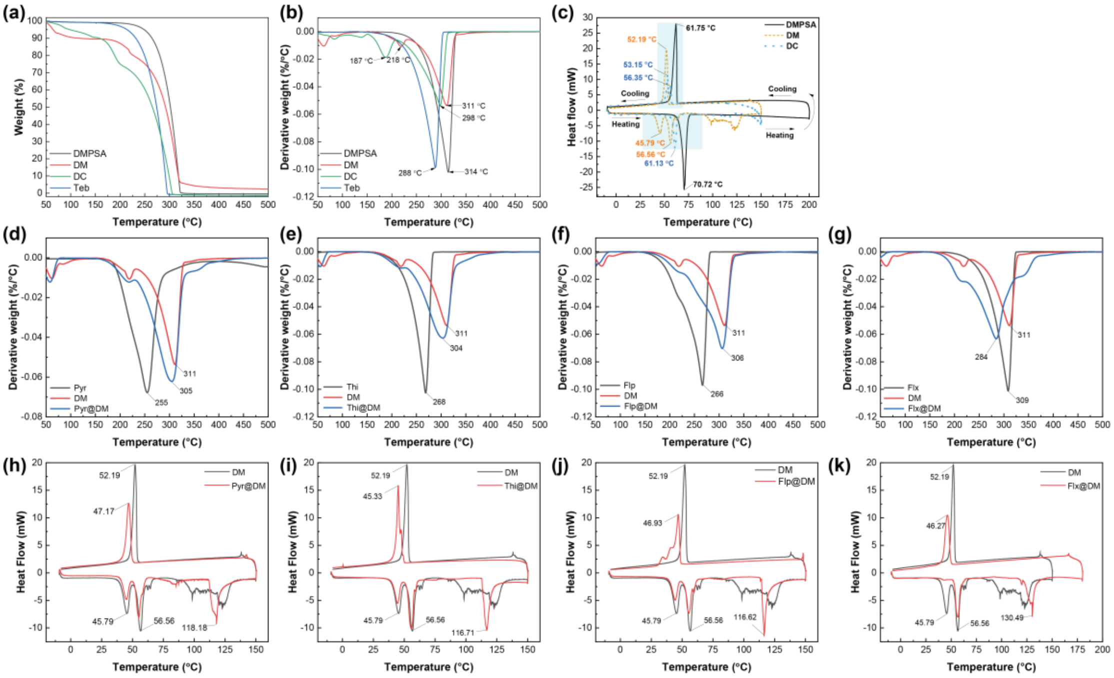

Figure S6. (a) TG and (b) DTG curves of DMPSA, Teb, and supramolecular lyophilized gels (DM and DC) within the temperature range of 50-500 °C, (c) DSC thermograms of DMPSA, and supramolecular lyophilized gels (DM and DC), (d-g) DTG curves of Pyr@DM, Thi@DM, Flp@DM, Flx@DM, (h-k) DSC thermograms of Pyr@DM, Thi@DM, Flp@DM, Flx@DM.

S2.3. Chemical Interaction Analysis

Fourier-transform infrared spectroscopy (FTIR) was utilized to analyze the interactions and track changes within the supramolecules, as depicted in Figures 3f and S7a&b. DM spectrum shows a shoulder peak at 3313 cm^{-1} for N-H stretching vibrations in comparison with DMPSA. The peak for the in-plane bending vibration of the amide II band shifts from 1550 cm^{-1} to 1560 cm^{-1}, and the peak for the C-N stretching vibration of the amide III band red-shifts from 1260 cm^{-1} to 1306 cm^{-1}. These shifts are attributed to hydrogen bonding between the N-H groups of DMPSA and MA. The stretching vibration peaks of the tertiary amine groups in DMPSA are observed at 2815 cm^{-1} and 2764 cm^{-1}. Upon addition of MA, these peaks blue-shift significantly to 2764 cm^{-1} and 2720 cm^{-1}, with a broadening at 2720 cm^{-1} due to protonation of the tertiary amine by carboxyl groups, forming quaternary ammonium salts. Both DMPSA and DM

exhibit a peak at 1641 cm^{-1}, corresponding to the C=O stretching vibration in the amide group, with no shift indicating no interaction between MA and the C=O group. The original sharp N-H peak at 3319 cm^{-1} in the amino-amide is merged into a broader peak at 3428 cm^{-1} due to the presence of O–H in carboxylic acid. These observations confirm interactions between DMPSA and maleic acid, primarily through protonation of the tertiary amine and weaker hydrogen bonding from the amide group. Compared to DM, DC exhibits a broader peak at 3428 cm^{-1} due to the higher carboxyl content from CA. Notably, the quaternary ammonium peak in DC is broader than in DM, indicating stronger hydrogen bonding between DMPSA and CA.

To further investigate the hydrogen-bonding strength between the DM and DC systems, ^{1}H NMR analysis was conducted on the two substances and the results were shown in Figures 3g&h and S7c&d. The peak for the *N*-dimethyl group of DMPSA appears at a chemical shift of $\delta = 2.24$ ppm. After reacting with MA, it shifts downfield to 2.58 ppm, attributed to the deshielding effect of hydrogen bonding. After adding Teb, the hydroxyl proton of Teb shifts by 0.012 ppm, indicating some hydrogen bonds between Teb and the supramolecular system. In contrast, the downfield shift of the *N*-dimethyl group in DMPSA is significant, indicating it is the main hydrogen-bonding force in the system. The weak hydrogen bonding with the fungicide enhances system stability and improves Teb's water solubility.

Moreover, the addition of h-BN causes no obvious chemical shift in ^{1}H NMR, suggesting h-BN merely participating in hydrogen bonding in the system. As for DC, the chemical shift at position 6* ($\triangle\delta = 0.57$ ppm) is due to the hydrogen bond formed between the dimethylamine in DMPSA and CA, which is larger than the case of DM ($\triangle\delta = 0.34$), indicating stronger N-H hydrogen bonding in DC. This aligns with the results of TG and infrared structural characterization.

The minor downfield shift of the amide N-H proton indicates a certain degree of electron density reduction, mainly because the amide N-H proton in DMPSA forms a new N-H-O hydrogen bond with the carboxyl oxygen atom (-COO-) of citric acid. This phenomenon is not observed in DM, indicating that different acids have a significant impact on the construction of DMPSA supramolecular hydrogen bonds.

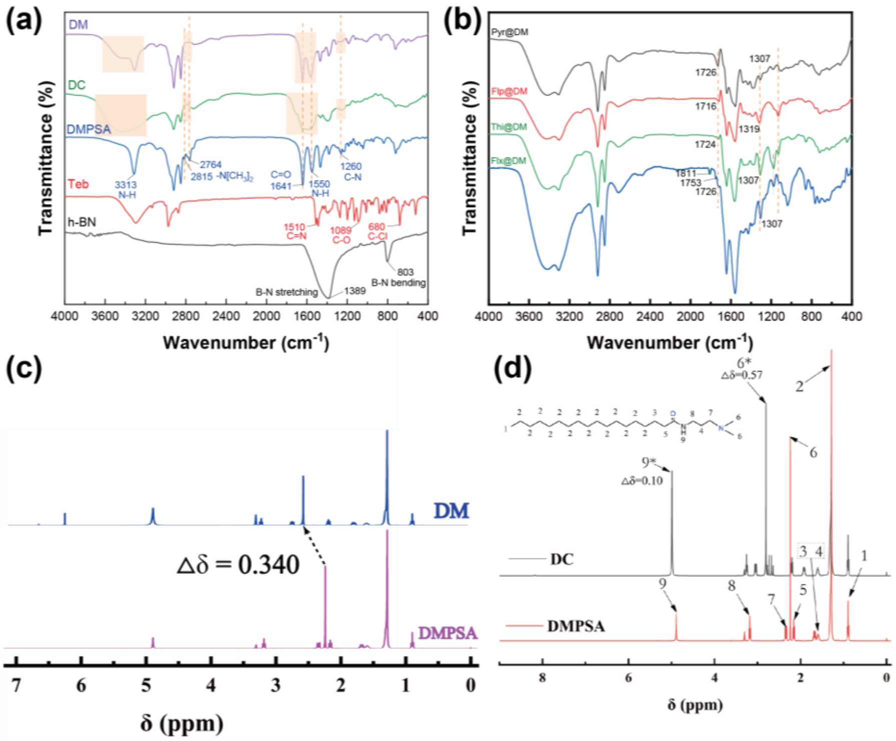

Figure S7. (a) FTIR spectra of DMPSA, DM, DC, pristine h-BN, and raw Teb in the range of 4000-400 cm^{-1}, (b) FTIR spectra of Pyr@DM, Thi@DM, Flp@DM, Flx@DM in the range of 4000-400 cm^{-1}, (c) ^{1}H NMR spectra of DMPSA and DM, (d) ^{1}H NMR spectra of DMPSA and DC.

XRD was employed to determine the crystallinity of h-BN within the gels and measure changes in its interlayer spacing, as illustrated in Figure S8. XRD patterns of the supramolecular gels in Figure S8a show characteristic peaks corresponding to the (002) crystal plane of hexagonal boron nitride, located between 26.68° and 26.74° in different freeze-dried gel samples. The assembling of Teb introduces new diffraction peaks. An interesting observation emerges from the XRD analysis of h-BN nanosheets isolated from the gels in Figure S8b. In detail, for h-BN separated from the fungicide-free gels (DM-hBN and DC-hBN), the (002) diffraction peak appears at 26.62°, corresponding to an interlayer spacing of 0.33 nm. In contrast, for h-BN isolated from the tebuconazole assembled gels (Teb@DM-hBN and Teb@DC-hBN), the diffraction angle shifts to a lower value of 26.42°, indicating an increased interlayer spacing of 0.34 nm.

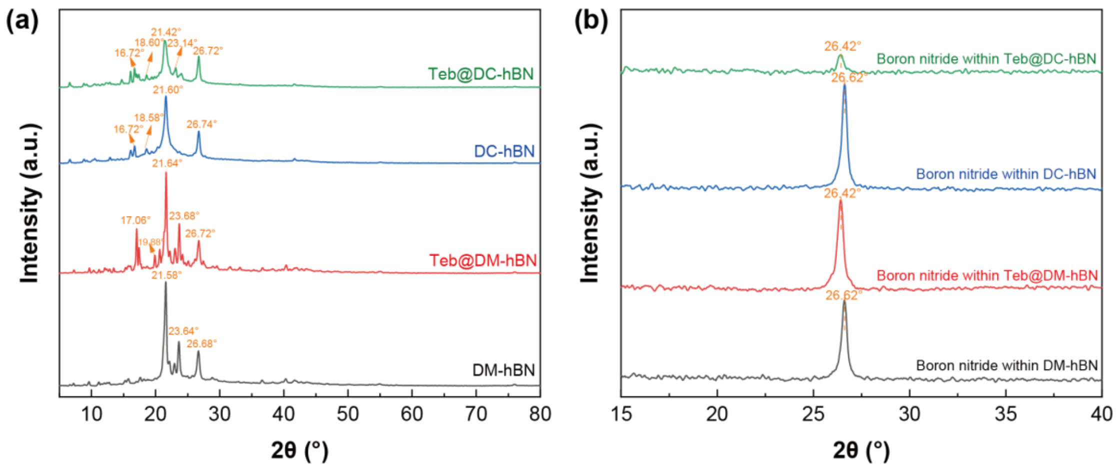

Figure S8. XRD patterns of (a) DM-hBN, Teb@DM-hBN, DC-hBN, Teb@DC-hBN after freeze drying, (b) corresponding boron nitride nanosheets collecting within DM-hBN, Teb@DM-hBN, DC-hBN, Teb@DC-hBN after washing and centrifugation.

XPS was used to compare and analyze the structural information of DMPSA and DM, as shown in Figures S9a&b. DMPSA only has one peak at 398.07 eV, which is attributed to N-C=O, C-N, and N-H bonds, indicating that the nitrogen in DMPSA is mainly in this chemical environment. In the N 1s spectrum of DM, the main peak is still at 399.00 eV, which is attributed to N-C=O, C-N, and N-H, accounting for 86.65%. An additional peak appears at 401.36 eV, which is attributed to $(NR_3H)^+$, accounting for 13.35%, indicating that some nitrogen in DMPSA is protonated. The amine groups in DM hydrogen bond with MA, resulting in the protonation of some amine groups, thereby generating peaks with higher binding energy. This change in chemical shift proves that MA interact with DMPSA.

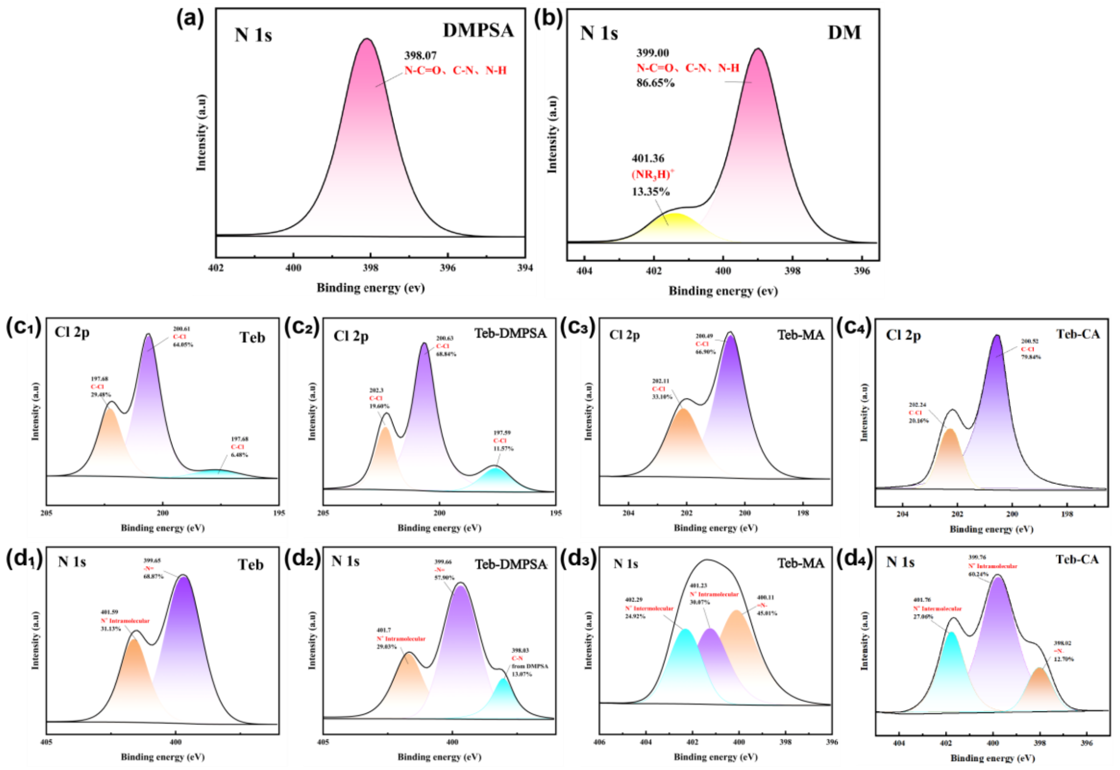

Figure S9. XPS spectra of (a) N 1s for DMPSA, (b) N 1s for DM, (c_1-c_4) Cl 2p for Teb, Teb-DMPSA, Teb-MA, Teb-CA, and (d_1-d_4) N 1s for Teb, Teb-DMPSA, Teb-MA, Teb-CA.

S2.4. The Assembly Mechanism

The assembly mechanism was investigated through a multi-faceted experimental approach, and the relevant data are presented in the main text and supporting information. We have now reorganized and expanded the discussion in the manuscript to highlight the following key evidences, which collectively substantiate the proposed mechanism as follows.

Protonation and ionic bridge formation: The evolution of solution conductivity and zeta potential during gel formation (discussed in Section 2.4) directly confirms the proton transfer from the polyprotic acids (citric/maleic acid) to the amino-amide gelator. This establishes the primary electrostatic driving force and the formation of ionic bridges, which are critical for initiating the self-assembly.

Molecular level interactions (discussed in Section 2.5&S2.3): The shifts in FTIR characteristic peaks (e.g. C=O stretching of amide and carboxylate groups, N-H bending) provide direct evidence for the formation of hydrogen bonds and electrostatic

interactions between the carboxylate anions and the protonated ammonium groups within the gel network. The significant chemical shift changes within ^{1}H NMR observed for the amide N-H and alkyl chain protons upon gelation further corroborate the involvement of hydrogen bonding and the changes in the local chemical environment due to molecular packing. The changes in the binding energies of N 1s and O 1s by XPS curves core levels offer surface sensitive confirmation of the electrostatic interaction and hydrogen bonding between the amino-amide and the acids.

Supramolecular structure and packing: The XRD patterns (discussed in Section 2.5&S2.3) reveal the crystalline nature of the assembly. The characteristic peaks and calculated d-spacings are consistent with a layered supramolecular structure, which is formed through the orderly packing of the gelators facilitated by the aforementioned non-covalent interactions.

S2.5. Release Kinetics of Teb from Supramolecular Gels with Respect to pH and Temperature Stimuli

In order to further investigate the release kinetics of Teb from the supramolecular system, data from the release curves was fitted by four diffusion models, including Logistic, First-order, Pseudo second-order, and Higuchi, as presented in Figure S10a-d and Table S2. The fitting results reveal that the release of Teb from Teb@DM and Teb@DM-hBN aligns closely with the Logistic and Pseudo second-order models under different pH conditions, as indicated by the high regression coefficients (R^2). This suggests that the release is governed by diffusion and chemical adsorption, with complex interplay chemical interactions and interfacial reactions between active ingredient and carrier, predominantly driven by hydrophobic and hydrogen bonding interactions. Similarly, the temperature-dependent release curves of the supramolecular delivery system were also analyzed using four kinetic models, as displayed in Figure S10e-h and Table S3. Among these, the Logistic model exhibits the best fit with highest regression coefficients, implying that the controlled release of Teb from DM and DM-hBN at different temperatures is primarily governed by a combination of diffusion and erosion mechanisms.

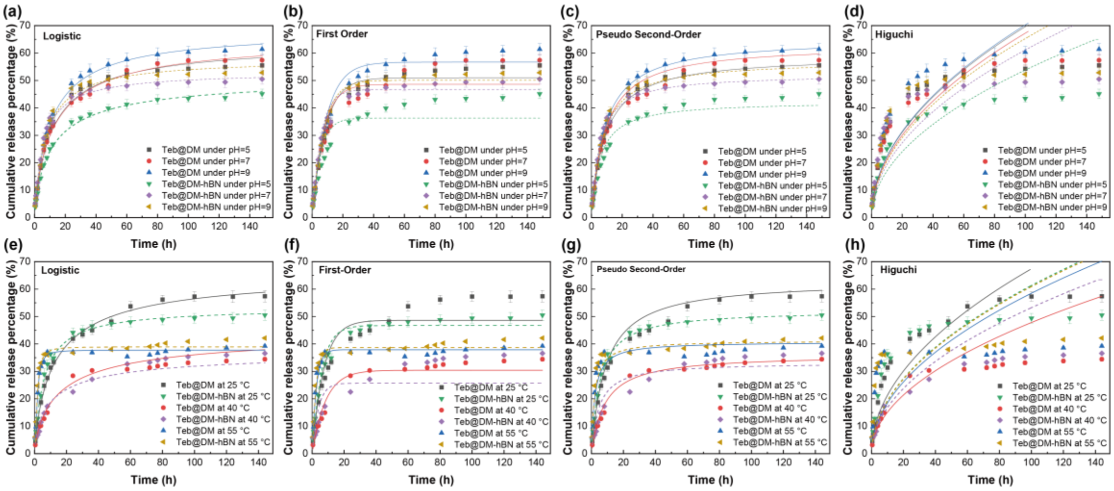

Figure S10. (a) pH-responsive release kinetic model fitting files of Teb@DM and Teb@DM-hBN under different pH conditions and (b) thermo-responsive release kinetic model fitting files of Teb@DM and Teb@DM-hBN under various temperature conditions fitting by Logistic, First-order, Pseudo second order, and Higuchi models.

Table S2. The fitting results of release curves for Teb@DM and Teb@DM-hBN under various pH conditions

Kinetic models	Fitting formula		Teb@DM				Teb@DM-hBN			
		pH	k_1	k_2	k_3	R^2	k_1	k_2	k_3	R^2
Logistic	$y = \frac{k_1 * exp\,[k_2 + k_2 * log\,(x)]}{1 + exp\,[k_2 + k_3 * log\,(x)]}$	5	64.294	2.202	0.896	**0.986**	52.261	1.972	0.796	**0.998**
		7	66.767	2.12	0.829	**0.997**	53.344	1.661	0.958	**0.997**
		9	68.769	2.149	0.918	**0.996**	58.006	1.948	0.982	**0.995**
First-order	$y = k_1[1 - exp\,(-k_2 x)]$	5	51.01	0.117		0.974	36.287	0.185		0.936
		7	48.626	0.137		0.961	46.717	0.173		0.986
		9	56.718	0.11		0.983	50.067	0.135		0.989
Pseudo second-order	$y = k_1 {k_2}^2 x/(1 + k_1 k_2\, x)$	5	0.002	59.155		**0.985**	0.004	42.446		0.98
		7	0.002	63.176		0.982	0.004	52.319		**0.996**
		9	0.002	65.336		0.995	0.002	57.405		**0.995**
Higuchi	$y = k_1 x^{1/2}$	5	6.962			0.873	5.328			0.905
		7	6.727			0.925	6.114			0.825
		9	7.026			0.891	6.529			0.852

Table S3. The fitting results of release curves for Teb@DM and Teb@DM-hBN at various temperature levels

Kinetic models	Fitting formula	T(°C)	Teb@DM k_1	Teb@DM k_2	Teb@DM k_3	Teb@DM R^2	Teb@DM-hBN k_1	Teb@DM-hBN k_2	Teb@DM-hBN k_3	Teb@DM-hBN R^2
Logistic	$y = \frac{k_1 * exp\,[k_2 + k_2 * log\,(x)]}{1 + exp\,[k_2 + k_3 * log\,(x)]}$	25	66.767	-2.12	0.829	**0.997**	53.344	-1.661	0.958	**0.997**
		40	45.235	-1.774	0.629	**0.992**	38.154	-0.793	1.886	**0.992**
		55	37.758	-1.043	1.981	**0.998**	38.975	-0.822	1.796	**0.991**
First-order	$y = k_1[1 - exp\,(-k_2x)]$	25	48.626	0.137		0.961	46.717	0.173		0.986
		40	30.375	0.119		0.925	25.708	0.261		0.878
		55	38.133	0.334		0.971	38.712	0.408		0.98
Pseudo second-order	$y = k_1{k_2}^2x/(1 + k_1k_2\,x)$	25	0.002	63.176		0.982	0.006	33.247		0.94
		40	0.003	36.125		0.951	0.006	35.155		0.924
		55	0.009	40.785		0.93	0.013	41.245		0.938
Higuchi	$y = k_1x^{1/2}$	25	6.727			0.925	6.114			0.905
		40	4.792			0.827	5.324			0.729
		55	7.063			0.399	6.113			0.038

S2.6. Wetting and Adhesion Characteristics on Foliar Surfaces

The comparative droplet impact experiments were conducted using both a pure water droplet and a droplet of the sol-state DM supramolecule on a hydrophobic PTFE surface. High-speed camera images captured at 2, 8, 16, and 32 ms after impact are presented in Figure S11. This experiment provides visual evidence that corroborates the decreasing contact angle trend in Figure 5. The water droplet retracts rapidly upon contacting the PTFE surface and reaches its maximum rebound height at 32 ms. In contrast, the DM supramolecular droplet exhibits immediate spreading and wetting behavior on the PTFE surface without rebounding after impact. The possible reason is that the supramolecular chains rapidly form intimate contact with the PTFE surface through hydrophobic interactions and van der Waals forces upon impact.

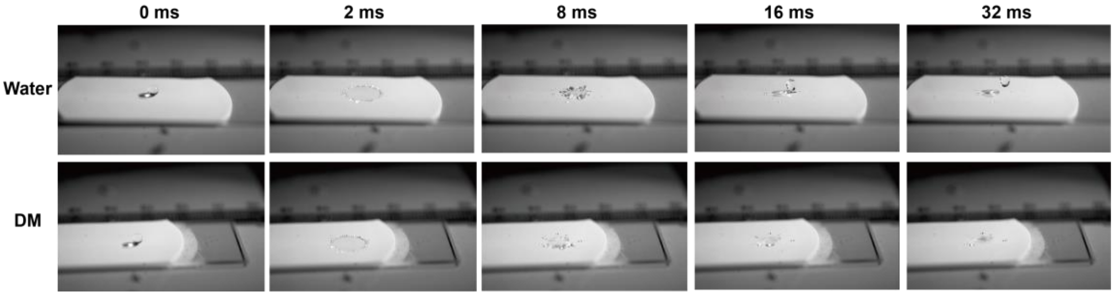

Figure S11. Snapshots of water and DM dynamic droplets bouncing on the PTFE membrane surface.

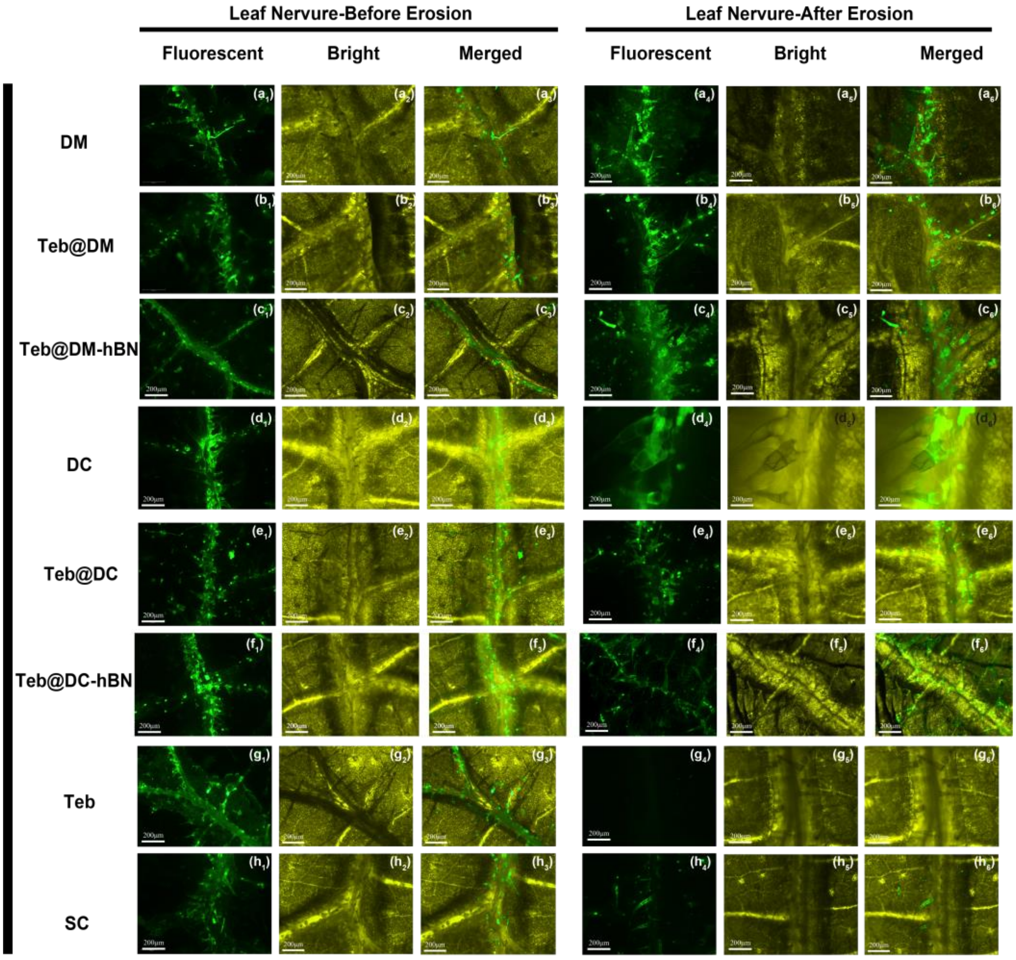

Figure S12. Inverted fluorescence images of cucumber leaf nervures domains rinsing with DM (a_1-a_3), Teb@DM (b_1-b_3), Teb@DM-hBN (c_1-c_3), DC (d_1-d_3), Teb@DC (e_1-e_3), Teb@DC-hBN (f_1-f_3), Teb (g_1-g_3), and SC (h_1-h_3), and the corresponding captured images after erosion.

S2.7. Supramolecules and Soil Interaction Analysis

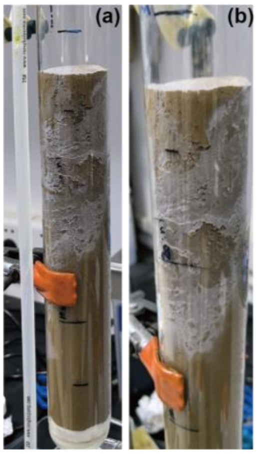

Figure S13. Digital photographs of soil column treated by (a) Teb@DC-hBN and (b) Teb@DM-hBN after leaching experiments, where the white traces around the outside of soils stemming from h-BN residue.

S2.8. Antifungal Activity *in Vitro*

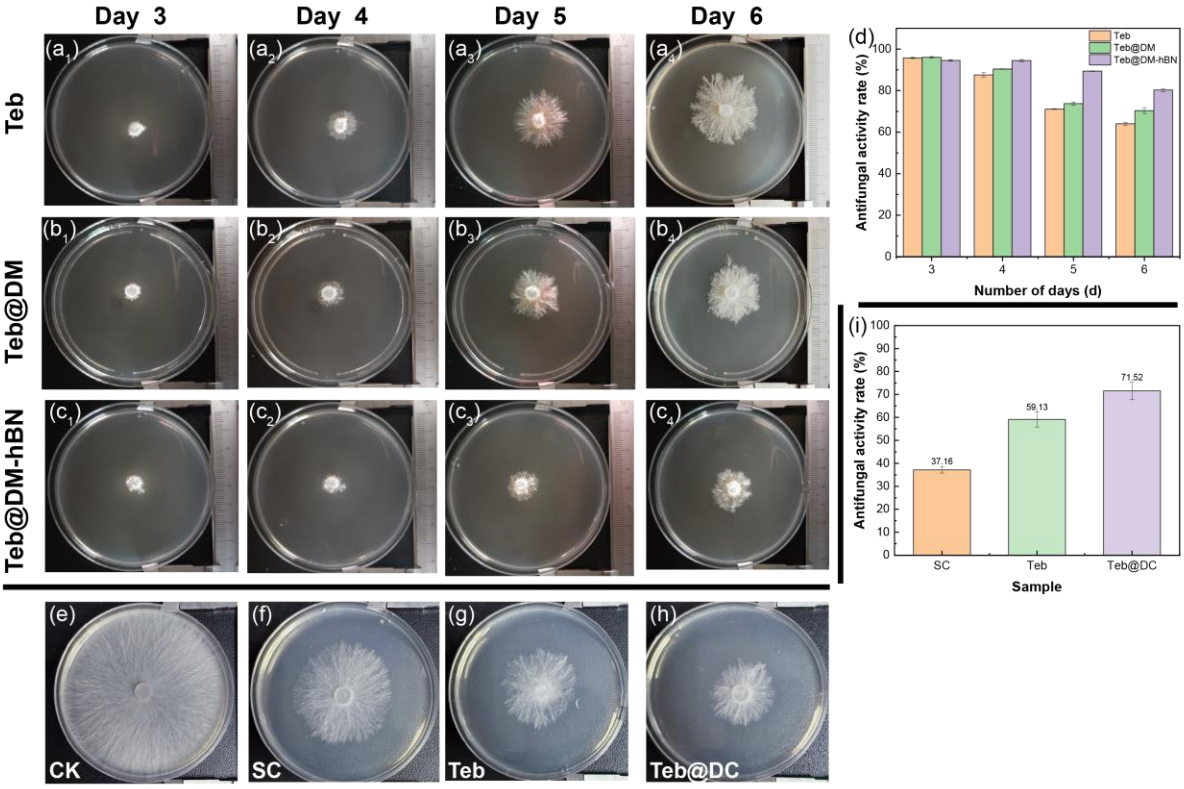

Figure S14. The digital images of the antifungal inhibition activity against *Sclerotium rolfsii* by Teb (a_1-a_4), Teb@DM (b_1-b_4), and Teb@DM-hBN (c_1-c_4) over time, (d) the histogram of the antifungal activity rates of Teb, Teb@DM, and Teb@DM-hBN over time, (e-h) the antifungal activity against *Sclerotium rolfsii* of CK, SC, Teb, Teb@DC, (i) the histogram of the antifungal activity rates of SC, Teb, Teb@DC.

Table S4. Effect concentration parameters of Teb, SC, Teb-loaded DM, DM-hBN, DC, DC-hBN.

Sample	EC_{50} (mg·L^{-1})	EC_{95} (mg·L^{-1})	95% Confidence interval	R^2
SC	0.059	0.412	0.047-0.075	0.964
Teb	0.061	0.389	0.049-0.076	0.978
Teb@DM	0.033	0.194	0.023-0.044	0.973
Teb@DM-hBN	0.028	0.213	0.019-0.041	0.978
Teb@DC	0.053	0.471	0.044-0.063	0.978
Teb@DC-hBN	0.044	0.364	0.036-0.053	0.965

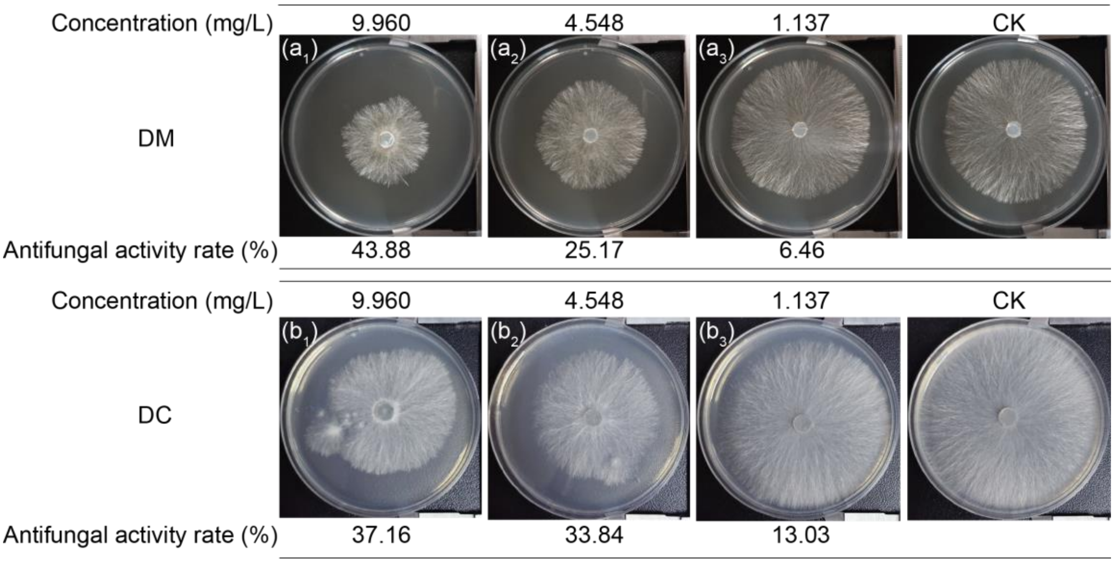

Figure S15. The digital images of the inhibition activity and antifungal activity rate against *Sclerotium rolfsii* by DM (a_1-a_3) and DC (b_1-b_3) at various Teb concentrations.

S2.9. Discussion on the Soil Environmental Safety

The supramolecular system was consciously constructed using building blocks derived from renewable resources, emphasizing sustainability and reduced environmental impact. The amino-amide component is usually synthesized from stearic acid (a common long-chain fatty acid found in nature) and *N*,*N*'-dimethyl-1,3-propanediamine via a condensation reaction.[1,4] Fatty acids are attractive precursors due to their low toxicity, ready biodegradability, low cost, and ease of functionalization, making them ideal for building sustainable and renewable supramolecular systems.[5-7] The resulting gelator is then self-assembled with citric acid or maleic acid. Both acids are natural or benign organic molecules. Citric acid is a ubiquitous natural metabolite, and maleic acid/hydrolyzed polymaleic anhydride has been studied and applied as an

environmentally friendly soil amendment in the literature without adverse effects.[8-10] The supramolecular network is held by non-covalent interaction, which are dynamic and susceptible to breakdown under environmental stressors. Consequently, the gel matrix is composed of components with a high likelihood of undergoing eventual disintegration during long-term period into harmless substances after fulfilling its function, rather than persisting indefinitely in the environment.

Hexagonal boron nitride is known for its high chemical inertness and thermal stability, similar to graphite.[11] Importantly, h-BN is widely reported to exhibit excellent biocompatibility and low toxicity.[12,13] After the gradual degradation of the supramolecular gel matrix, the h-BN nanosheets will persist in the soil but likely as an inert, non-toxic residue, much like natural clay platelets. Their potential interaction with the soil ecosystem is an important area for future long-term studies, but current evidence suggests a favorable safety profile.

The primary environmental benefit of this system lies in dramatically reducing tebuconazole dosage and minimizing leaching, which presents a significant environmental advantage over conventional application methods.

References

[1] Li, H.; Dandan, H.; Mei, G. Preparation of supramolecular-assemble hydrogels as pesticide carriers based on amphiphilic amino-amide compounds. *CIESC Journal* 2020, 71 (8), 3819-3829.

[2] X. Li, N. Tao, B. Xu, J. Xu, Z. Yang, C. Jiang, Y. Zhou, M. Deng, J. Lv, K. Zhao, Establishment and application of a root wounding–immersion method for efficient virus-induced gene silencing in plants. Frontiers in Plant Science 15 (2024) 1336726.

[3] W. Qin, H. Yan, B. Zou, R. Guo, D. Ci, Z. Tang, X. Zou, X. Zhang, X. Yu, Y. Wang, Arbuscular mycorrhizal fungi alleviate salinity stress in peanut: evidence from pot-grown and field experiments, *Food and Energy Security* 10(4) (2021) e314.

[4] L. Hao, C. Yegin, J.V. Talari, J.K. Oh, M. Zhang, M.M. Sari, L. Zhang, Y. Min, M. Akbulut, B. Jiang, Thermo-responsive gels based on supramolecular assembly of an amidoamine and citric acid, *Soft Matter* 14(3) (2018) 432-439.

[5] S. Wang, S. V. Kesava, E. D. Gomez, M. Robertson, Sustainable thermoplastic elastomers derived from fatty acids, *Macromolecules* 46 (18) (2013) 7202-7212.

[6] A. L. Holmberg, K. H. Reno, R. P. Wool, T. H. Epps, Biobased building blocks for the rational design of renewable block polymers, *Soft Matter*, 10 (38) (2014) 7405-7424.

[7] R Vendamme, N Schüwer, W Eevers. Recent synthetic approaches and emerging bio-inspired strategies for the development of sustainable pressure-sensitive adhesives derived from renewable building blocks, *Journal of Applied Polymer Science*, 131 (17) (2014) 40669.

[8] H. Xu, S. Yan, E. Gerhard, D. Xie, X. Liu, B. Zhang, D. Shi, G.A. Ameer, J. Yang, Citric acid: a nexus between cellular mechanisms and biomaterial innovations, *Advanced Materials* 36(32) (2024) 2402871.

[9] G. Cooper, C. Reed, D. Nguyen, M. Carter, Y. Wang, Detection and formation scenario of citric acid, pyruvic acid, and other possible metabolism precursors in carbonaceous meteorites, *Proceedings of the National Academy of Sciences* 108(34) (2011) 14015-14020.

[10] E. Asghari, M. Gholizadeh-Khajeh, H. Ashassi-Sorkhabi, Tartaric acid as a non-toxic and environmentally-friendly anti-scaling material for using in cooling water systems: electrochemical and surface studies, *Journal of Materials Engineering and Performance* 25(10) (2016) 4230-4238.

[11] Q. Weng, X. Wang, X. Wang, Y. Bando, D. Golberg, Functionalized hexagonal boron nitride nanomaterials: emerging properties and applications, *Chemical Society Reviews* 45(14) (2016) 3989-4012.

[12] M. Anas, B. Fatima, Z. Zahid, N. Waqar, Applications of 2D Materials in Agriculture, 2D Materials: Fundamentals, Fabrication, and Applications, Springer2025, pp. 281-318.

[13] S.M. Sharker, Hexagonal boron nitrides (white graphene): a promising method for cancer drug delivery, *International Journal of Nanomedicine* 14 (2019) 9983.
